# Integrative Metabolomic, Network Pharmacology, and Experimental Evidence for *Lepidium sativum* Seed Extract as a Natural Modulator of Pulmonary Fibrosis via the *ncNRFR/Let-7d* Regulatory Pathway

**DOI:** 10.3390/ph18121820

**Published:** 2025-11-28

**Authors:** Ibrahim M. Alanazi, Hebatallah H. Abo Nahas, Doaa I. Mohamed, Nora Hosny, Yaser H. A. Elewa, Manisha Agarwal, Ibrahim Abdel Aziz Ibrahim, Alaa Hisham Falemban, Ghazi A. Bamagous, Emad Rashad Sindi, Tarek A. Yousef, Sanchaita Rajkhowa, Maha Alsunbul, Essa M. Saied

**Affiliations:** 1 Department of Pharmacology and Toxicology, Faculty of Medicine, Umm Al-Qura University, Makkah 77207, Saudi Arabia; imanzi@uqu.edu.sa (I.M.A.); iamustafa@uqu.edu.sa (I.A.A.I.); ahfalemban@uqu.edu.sa (A.H.F.); gabamagous@uqu.edu.sa (G.A.B.); 2 Zoology Department, Faculty of Science, Port Said University, Port Said 42526, Egypt; heba.hassan@sci.psu.edu.eg; 3 Department of Clinical Pharmacology and Therapeutics, Faculty of Medicine, Ain Shams University, Cairo 11566, Egypt; doaapharma@med.asu.edu.eg; 4 Medical Biochemistry and Molecular Biology Department, Faculty of Medicine, Suez Canal University, Ismailia 41522, Egypt; nora_hosny@med.suez.edu.eg; 5 Department of Histology, Faculty of Veterinary Medicine, Zagazig University, Zagazig 44511, Egypt; y-elewa@vetmed.hokudai.ac.jp; 6 Faculty of Veterinary Medicine, Hokkaido University, Sapporo 0600818, Japan; 7 Centre for Biotechnology and Bioinformatics, Dibrugarh University, Dibrugarh 786004, India; manishaagarwal1022@gmail.com (M.A.); s_rajkhowa@dibru.ac.in (S.R.); 8 Department of Basic Medical Sciences, College of Medicine, University of Jeddah, Jeddah 23890, Saudi Arabia; ersindi@uj.edu.sa; 9 Chemistry Department, College of Science, Imam Mohammad Ibn Saud Islamic University (IMSIU), Riyadh 11623, Saudi Arabia; tayousef@imamu.edu.sa; 10 Department of Pharmaceutical Sciences, College of Pharmacy, Princess Nourah Bint Abdulrahman University, P.O. Box 84428, Riyadh 11671, Saudi Arabia; maalsonbel@pnu.edu.sa; 11 Chemistry Department, Faculty of Science, Suez Canal University, Ismailia 41522, Egypt; 12 Institute for Chemistry, Humboldt Universität zu Berlin, 12489 Berlin, Germany

**Keywords:** pulmonary fibrosis, *Lepidium sativum*, phytochemical profiling, antioxidant activity, network pharmacology, *ncNRFR/let-7d* modulation, histopathology

## Abstract

**Background/Objectives**: Pulmonary fibrosis (PF) is a progressive interstitial lung disease with limited therapeutic options. *Lepidium sativum* (cress seeds) possess recognized antioxidant and anti-inflammatory properties, yet its potential antifibrotic activity has not been investigated. This study evaluated the phytochemical composition and antifibrotic efficacy of cress seed extract (CSE) and examined whether its effects are associated with modulation of the *ncNRFR/let-7d* pathway in methotrexate (Mtx)-induced PF. **Methods**: Comprehensive metabolite profiling was performed using GC–MS, HPLC, and UPLC–T-TOF–MS/MS. Antioxidant capacity and antiproliferative effects were assessed in vitro. Network pharmacology was used to identify CSE-related PF targets and regulatory pathways. In vivo, PF was induced in adult male Wistar rats by Mtx, followed by oral CSE administration (50–150 mg/kg). Biochemical markers of inflammation, oxidative stress, extracellular matrix deposition, EMT, and ncRNA expression (*ncNRFR* and *let-7d*) were quantified alongside histopathology and immunohistochemistry. **Results**: CSE contained diverse terpenes, phenolics, flavonoids, glucosinolates, and amino acid derivatives. It exhibited potent antioxidant activity and antiproliferative effects against A549 and Hep2 lung cancer cells. Network analysis identified 997 overlapping CSE–PF targets and highlighted IL6 and MMP1 as relevant *miR-let-7d*–associated nodes. In vivo, Mtx-induced marked fibrosis characterized by increased *ncNRFR*, reduced *let-7d*, elevated IL6, HMGB1, TGF-β, MMP1, collagen, and hydroxyproline, and reduced antioxidant enzyme activity. CSE treatment dose-dependently mitigated these alterations, improved histoarchitecture, and reduced collagen deposition. **Conclusions**: CSE showed antifibrotic, antioxidant, and anti-inflammatory activity in MTX-induced PF in rats and modulated the reciprocal expression patterns of *ncNRFR* and *let-7d*. These findings support CSE as a potential source of bioactive constituents for PF management and identify the putative *ncNRFR–let-7d* regulatory relationship as a novel pathway in fibrotic lung disease, warranting further mechanistic investigation.

## 1. Introduction

Pulmonary fibrosis (PF) is a chronic interstitial lung disease with an estimated incidence of ~5.6 cases per 100,000 individuals annually [1,2]. It is characterized by progressive fibrosis, inflammation, and structural remodeling of the lung, ultimately leading to impaired gas exchange and respiratory failure [3]. A hallmark of PF is the excessive accumulation of extracellular matrix (ECM) proteins in the lung interstitium, which disrupts normal parenchymal architecture and drives functional decline [4]. Within the spectrum of interstitial lung diseases (ILD), idiopathic pulmonary fibrosis (IPF) represents the most severe form, with a particularly poor prognosis and high mortality [5]. The precise cellular and molecular mechanisms underlying PF remain incompletely understood. Aberrant alveolar epithelial injury and impaired repair responses are thought to play central roles in initiating fibrotic remodeling. Although the origin of pathogenic myofibroblasts is debated, accumulating evidence implicates epithelial–mesenchymal transition (EMT) as a key process contributing to fibroblast accumulation in fibrotic tissue [6].EMT is a biological program in which epithelial cells lose polarity and cell–cell adhesion, while acquiring mesenchymal characteristics such as motility, invasiveness, and expression of fibroblast-like markers [7,8,9]. This transition promotes matrix deposition and structural distortion, thereby accelerating the progression of PF.

The *let-7* family was first discovered in *C. elegans* as a regulator of developmental timing and later became the first miRNA identified in humans. Its family members are highly conserved across species, underscoring their fundamental role in controlling cell proliferation and differentiation [10]. Dysregulation of *let-7* expression has been linked to cellular dedifferentiation and the pathogenesis of multiple diseases [11]. Among the *let-7* isoforms, *let-7d* has been particularly downregulated in idiopathic pulmonary fibrosis (IPF), leading to the overexpression of profibrotic mediators such as HMGB1, MMP1, and IL16, with consequent collagen deposition and excessive EMT in mouse lungs [12]. Experimental inhibition of let-7d further increased mesenchymal markers while reducing epithelial markers, consistent with EMT activation in fibrotic lungs [13]. Mechanistically, *let-7d* appears to regulate E-cadherin, a classical cadherin essential for epithelial cell–cell adhesion, embryonic development, and tissue integrity [14]. Through its cytoplasmic interactions with the cytoskeleton, E-cadherin influences key pathways including Hippo, Wnt, TGF-β, and NF-κB. Importantly, reduced let-7 expression has been associated with suppressed E-cadherin, loss of epithelial integrity, and promotion of EMT, whereas upregulation of *let-7* enhances E-cadherin expression and helps maintain epithelial barrier function [15,16]. Thus, dysregulation of the *let-7*/E-cadherin axis represents a critical mechanism in the progression of pulmonary fibrosis. Recent studies have highlighted the importance of non-coding RNAs (ncRNAs), a diverse class of transcripts that, although not translated into proteins, play critical roles in cellular and physiological regulation. Among them, long non-coding RNAs (lncRNAs), transcripts longer than 200 nucleotides, are involved in diverse processes including cell proliferation, development, apoptosis, and metastasis [17,18]. LncRNAs often act as competing endogenous RNAs (ceRNAs), sequestering specific miRNAs and thereby modulating the expression of their downstream targets [19]. Through interactions with RNA, DNA, and proteins, lncRNAs regulate gene transcription and translation by functioning as guides, scaffolds, or decoys that influence chromatin structure, mRNA stability, and protein recruitment [20,21]. One such lncRNA, ncNRFR (non-coding Nras functional RNA), was shown to attenuate the function of miRNA *let-7* by binding to its recognition elements, thereby reducing its regulatory effect on endogenous mRNAs [22,23]. Given the established role of *let-7d* in pulmonary fibrosis, dysregulation of *ncNRFR* may represent a novel regulatory mechanism contributing to disease progression.

The lung scarring associated with pulmonary fibrosis is largely irreversible, and, to date, no curative therapy has been identified. Current treatments are mainly palliative, aiming to slow disease progression or alleviate symptoms rather than achieve full resolution. The antifibrotic agents pirfenidone and nintedanib have demonstrated the ability to slow disease progression; however, their clinical benefits are often offset by adverse effects such as fatigue, gastrointestinal intolerance, and hepatotoxicity [3,24,25]. Thus, the identification of novel therapeutic strategies that effectively target fibrotic remodeling while minimizing toxicity remains a pressing challenge. Cress seeds (CS; *Lepidium sativum*) have a long history of use in traditional medicine, particularly for the management of inflammatory conditions, respiratory disorders, and digestive ailments [26,27,28,29]. Beyond their culinary applications, CS are valued for their rich nutrient content, including vitamins A, C, and K, calcium, and iron. Phytochemical investigations have revealed a wide spectrum of secondary metabolites, phenolics, flavonoids, coumarins, alkaloids, triterpenes, tannins, sterols, sinapic acid, glucosinolates, and sulforaphane derivatives, which likely contribute to their bioactivity [27,28,29]. Consistent with this composition, CS extracts have demonstrated diverse pharmacological effects, including antihypertensive, antiasthmatic, hepatoprotective, immunomodulatory, antioxidant, anti-inflammatory, and hypoglycemic properties [30]. Of particular relevance, the ethanolic extract of CS was shown to attenuate liver fibrosis by inhibiting fibroblast activation, reducing collagen accumulation, and limiting ECM protein deposition, highlighting both antioxidant and antifibrotic mechanisms [31]. These findings suggest that CS phytochemicals may hold therapeutic value in fibrotic disorders; however, their potential role in pulmonary fibrosis has not yet been systematically explored.

Based on these considerations, and in continuation of our efforts to explore novel pharmacological agents [32,33,34,35], the present study aimed to evaluate, for the first time, the therapeutic potential of cress seed extract (CSE) in mitigating pulmonary fibrosis. We first characterized the metabolomic profile of CSE using HPLC, GC–MS, and UPLC–T-TOF–MS/MS to identify its phytochemical repertoire. Next, we assessed its antiproliferative and antioxidant activities through in vitro assays. To extend these findings and uncover molecular mechanisms, we employed network pharmacology, integrating CSE-derived targets with fibrosis-related genes. Protein–protein interaction analysis identified hub genes linked to fibrosis, inflammation, and oxidative stress, while enrichment analysis confirmed their involvement in profibrotic pathways. In parallel, lncRNA–miRNA–mRNA interaction analysis highlighted the regulatory significance of the *ncNRFR* and *let-7d* in modulating these pathways. Finally, we validated these predictions in vivo by examining the antifibrotic effects of CSE in the methotrexate-induced lung fibrosis model, combining molecular, biochemical, histological, and immunohistochemical analyses. In this regard, Wistar rats were selected due to their well-characterized pulmonary structure and reproducible response to fibrogenic stimuli. The methotrexate (Mtx) model was chosen because Mtx induces marked oxidative stress, inflammation, and extracellular matrix deposition that closely parallel key pathogenic features of human pulmonary fibrosis. This model provides a robust and reproducible platform for evaluating antifibrotic candidates and has been widely used in preclinical studies investigating lung injury [36,37,38]. Collectively, this study provides the first evidence that CSE exerts antifibrotic activity by targeting the *ncNRFR/let-7d* regulatory pathway and modulating key inflammatory, fibrotic, and antioxidant pathways in pulmonary fibrosis.

## 2. Results

### 2.1. Characterization of CSE Metabolic Profile

#### 2.1.1. Gas Chromatography-Mass Spectrometry (GC-MS) Analysis

**Figure 1:**
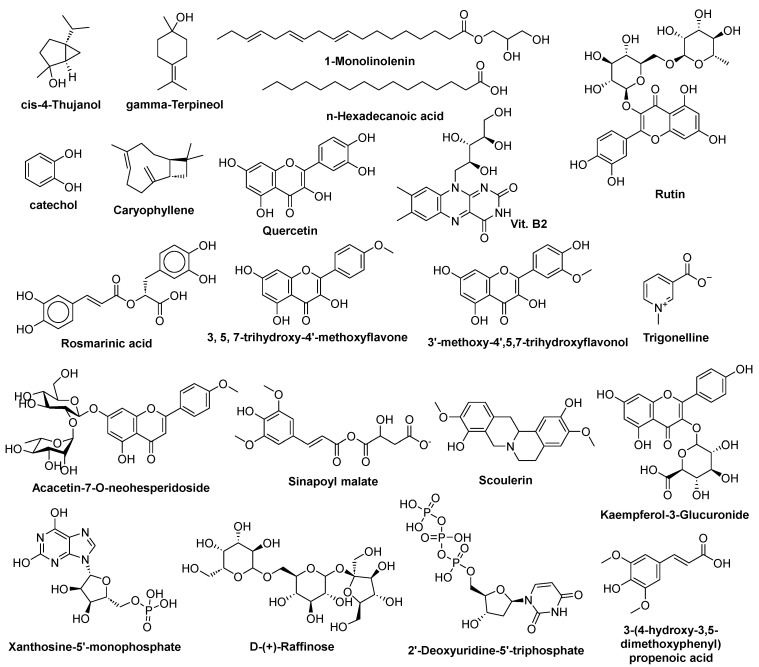
Structure of representative metabolites detected in the CSE by UPLC/T-TOF, GC-MS, and HPLC analysis.

GC–MS analysis of the methanolic extract of *CSE* identified 24 metabolites spanning seven major chemical classes: terpenoids, fatty acids and esters, neutral lipids, flavonoids, alkaloids, hydrocarbons, and sterols/phenolics (Table 1, Figures S1–S25). Terpenoids were the most abundant group, accounting for 48.38% of the total ion chromatogram (TIC) peak area. Within this class, monoterpenes were dominant (40.96%), with γ-terpineol (20.82%), cis-4-thujanol (13.20%), and cis-β-terpineol (3.21%) as key constituents. Sesquiterpenes (caryophyllene, γ-elemene) contributed 5.93%, while neophytadiene, a diterpene hydrocarbon, represented 1.49% (Figure 1). Fatty acids and esters were detected at 11.9%, primarily n-hexadecanoic acid (8.50%), methyl linoleate (1.30%), and methyl oleate (2.10%). Neutral lipids (13.49%) included 1-monolinolenin (10.80%) and trilinolein (1.55%). Two flavonoids (Lucenin II, 3′,4′,7-trimethoxyquercetin) comprised 3.44% of the extract, while alkaloids (dasycarpidan-1-methanol acetate, 10-methoxycoryn-18-en-17-yl acetate) accounted for 3.16%. Hydrocarbons (7.57%) were dominated by 3-ethyl-5-(2-ethylbutyl)-octadecane (5.51%). Other significant constituents included sterols (stigmast-5-en-3-ol, 1.90%) and phenolics (Isochiapin B, ±-methoxyphenylacetic acid, 8.46% combined). The metabolite profile indicates that *CSE* contains multiple classes of phytochemicals.

#### 2.1.2. High-Performance Liquid Chromatography (HPLC) Analysis

To complement the GC–MS profiling, a targeted HPLC analysis was performed to quantify key polar, thermolabile metabolites, including flavonoids, phenolic acids, and water- and fat-soluble vitamins in the methanolic extract of *CSE*. A total of 20 metabolites were detected and quantified (Table S1, Figure S26). Among these, phenolics were the most abundant group, with a total concentration of 40.14 µg/mL, followed by water-soluble vitamins (WSV, 35.76 µg/mL), flavonoids (31.97 µg/mL), and fat-soluble vitamins (FSV, 30.51 µg/mL). Within the phenolic acids, catechol (14.52 µg/mL) and syringic acid (10.69 µg/mL) were the major constituents, alongside cinnamic (6.05 µg/mL), caffeic (5.12 µg/mL), salicylic (2.44 µg/mL), and ferulic acid (1.32 µg/mL). The flavonoid fraction was dominated by quercetin (13.07 µg/mL) and rutin (10.48 µg/mL), with luteolin (3.08 µg/mL), apigenin (2.13 µg/mL), naringin (1.88 µg/mL), and kaempferol (1.33 µg/mL) detected in lower amounts (Figure 1). The vitamin profile included five WSV compounds: vitamin B2 (riboflavin, 10.16 µg/mL), B3 (niacin, 9.69 µg/mL), B6 (pyridoxine, 8.36 µg/mL), B1 (thiamine, 6.14 µg/mL), and vitamin C (ascorbic acid, 1.41 µg/mL). Three FSV compounds were also quantified, notably vitamin K (12.55 µg/mL), vitamin D (9.22 µg/mL), and retinol (8.74 µg/mL). Overall, catechol, syringic acid, rutin, quercetin, vitamin B2, and vitamin K emerged as the most abundant metabolites (≥10 µg/mL). Several metabolite classes overlapped between GC–MS and HPLC analyses, such as flavonoids and fatty acids, demonstrating the robustness of the chemical profile, while other compounds, including vitamins and highly polar phenolics, were uniquely detected via HPLC due to their poor volatility. Conversely, GC–MS revealed abundant terpenoids and hydrocarbons not captured in HPLC. Together, these techniques offer a comprehensive characterization of the metabolic profile of CSE.

#### 2.1.3. Untargeted UPLC–ESI–QTOF–MS Analysis

Untargeted UPLC–ESI–QTOF–MS profiling (ESI+) of the methanolic extract of CSE annotated 55 metabolites spanning seven chemical classes (by relative total peak area, TPA%, Table 2). The dataset was dominated by amino acids and derivatives (6 metabolites; 43.08% TPA), carbohydrates (4 metabolites; 26.20%), flavonoids (18 metabolites; 11.10%), nucleosides/nucleotides (9 metabolites; 10.64%), organic acids and phenylpropanoids (6 metabolites; 4.60%), alkaloids (2 metabolites; 2.16%), and other phenolics/amines (10 metabolites; 1.66%) (Figure S27). Within amino acids and derivatives, N,N-dimethylglycine was the major feature (34.94% TPA), with L-tryptophan (6.03%) and glycine betaine (1.58%) also contributing appreciably. Among carbohydrates, melibiose (17.43%) and maltotriose (7.40%) were most abundant. The flavonoid pool comprised aglycones (n = 11) and O-glycosides (n = 7); key aglycones included 3′-methoxy-4′,5,7-trihydroxyflavonol (3.62%) and 3,5,7-trihydroxy-4′-methoxyflavone (3.04%), while representative glycosides were acacetin-7-O-neohesperidoside (0.97%) and kaempferol-3-O-α-L-rhamnoside (0.69%). The nucleosides/nucleotides class (10.64% TPA) was led by 2′-deoxycytidine (4.10%), xanthosine-5′-monophosphate (3.80%), and adenosine (0.90%). Organic acids and phenylpropanoids totaled 4.60%, with sinapoyl malate (3.02%) and nicotinic acid (0.60%) as the top contributors. Alkaloids were represented by scoulerine (benzylisoquinoline; 1.04%) and trigonelline (pyridine alkaloid; 1.10%). Other phenolics/amines accounted for 1.66% (e.g., 4-aminophenol, 0.50%). Percentages are reported as TPA% within the ESI+ dataset; minor deviations from 100% reflect rounding (Table S2).

On the other hand, the untargeted UPLC–ESI–QTOF–MS profiling (ESI–) of the methanolic extract of *CSE* annotated 50 metabolites across six chemical classes (reported as total peak area, TPA% within the ESI– dataset). The profile was dominated by carbohydrate-related metabolites (87.58% TPA; 8 metabolites), driven largely by benzyl glucosinolate (66.77%) together with the oligosaccharides, sucrose (15.22%) and D-(+)-raffinose (5.25%). Organic acids and phenylpropanoids (7.35% TPA; 15 metabolites) included sinapoyl malate (2.80%), sinapic acid (3-(4-hydroxy-3,5-dimethoxyphenyl)-2-propenoic acid; 1.40%), and rosmarinic acid (1.19%). The flavonoid fraction (2.60% TPA; 12 metabolites) comprised both aglycones and conjugates, led by 3′-methoxy-4′,5,7-trihydroxyflavonol (0.80%) and kaempferol-3-O-glucuronide (0.70%). Nucleosides/nucleotides (1.50% TPA; 7 metabolites) were headed by 2′-deoxyuridine-5′-triphosphate (dUTP) (0.60%). Amino acids and derivatives contributed 0.68% TPA (6 metabolites). Two other minor phenolics/coumarins summed to 0.11%. (Table S3). Consistent with ionization chemistry, ESI+ highlighted basic/zwitterionic metabolites (amino acids/derivatives, flavonoid aglycones, nucleosides), whereas ESI– accentuated anionic/conjugated species (phenylpropanoid acids and conjugates, flavonoid glucuronides, nucleotides, and glucosinolates), with limited overlap (e.g., sinapoyl malate), confirming complementary coverage of the CSE metabolome (Figure 1). Together, these datasets depict a metabolome rich in volatiles (terpenoids), polar antioxidants (phenolics/flavonoids/vitamins), and primary metabolism markers (amino-acid betaines, sugars, nucleotides), supporting the extract’s putative bioactivity profile (Figures S27 and S28).

### 2.2. In Vitro Evaluation of Cytotoxicity and Antioxidant Potential

#### 2.2.1. Evaluation of Antiproliferative Activity

**Figure 2:**
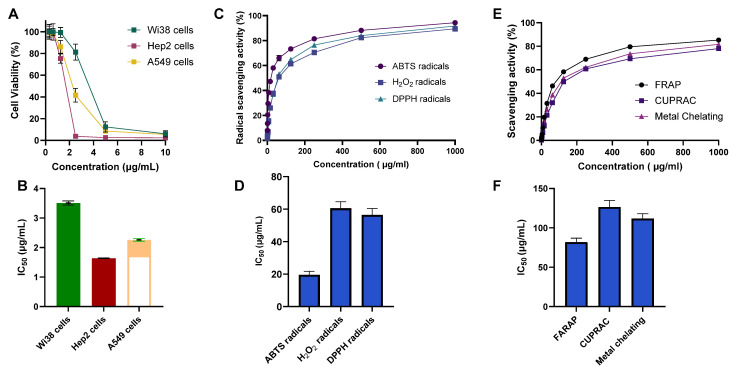
The in vitro antiproliferative and antioxidant activity of CSE. (**A**,**B**) The assessment of cell viability % of Wi38, Hep2, and A549 cells after treatment with CSE at different concentrations (**A**), and the corresponding diagram for the IC_50_ concentration of CSE toward the examined cells. (**C**–**F**) The in vitro antioxidant potential of CSE as examined by scavenging activity (%) toward ABTS, H_2_O_2_, and DPPH radicals (**C**,**D**), and copper and iron ions, and metal chelating (**E**,**F**). The presented data was expressed as mean ± S.D in triplicate.

We next envisioned in vitro examining the antiproliferative activity of CSE toward the lung cancer cells (Hep2 and A549 cells). In this regard, the cells were treated with various concentrations of CSE (0–10 µg/mL), and the viability of the cells was assessed utilizing the MTT assay. As shown in Figure 2, the CSE displayed substantial antiproliferative activity toward Hep2 and A549 cells in a dose-dependent manner. Interestingly, the assessment of IC_50_ values revealed that CSE exhibits significant cytotoxicity toward the examined Hep2 and A549 cells with IC_50_ of 1.44 µg/mL and 2.16 µg/mL, respectively (Figure 2A,B). These results suggest that CSE possesses a remarkable antiproliferative activity toward lung cancer cells. To further explore the selectivity and toxicity of CSE toward normal cells, we conducted an MTT assay utilizing the diploid human cells (Wi38 cells). CSE displayed antiproliferative activity toward Wi38 cells with an IC_50_ value of 3.81 µg/mL. These findings indicate that CSE exhibits a higher cytotoxicity toward the examined lung cancer cells (Hep2 and A549 cells) compared to Wi38 cells, although the selectivity indices were below accepted thresholds for defining meaningful selectivity, with a selectivity index of 2.66 and 1.8, respectively. Taken together, our investigations indicate that CSE exhibits significant antiproliferative activity toward lung cancer cells. Given that pulmonary fibrosis is associated with an elevated risk of lung carcinogenesis, the antiproliferative activity of CSE toward the lung adenocarcinoma A549 cell line may also represent a potentially beneficial characteristic.

#### 2.2.2. Evaluation of Antioxidant Activity

Furthermore, we evaluated the antioxidant potential of CSE using the total antioxidant capacity (TAC) assay. Our results revealed that CSE exhibits a TAC of 21.65 ± 0.91 mg Gallic acid equivalent (GAE)/g, compared to the reference ascorbic acid with a TAC of 74.92 ± 2.86 mg GAE/g. To profile specific mechanisms, we extended our examinations to explore the antioxidant activity of CSE to scavenge the free radicals and metal ions. As shown in Figure 2, our analysis revealed that CSE displays considerable scavenging activity toward the ABTS, H_2_O_2_, and DPPH radicals in a dose-dependent manner (Figure 2C,D). The assessment of the IC_50_ values revealed that CSE exhibits substantial scavenging activity toward ABTS, DPPH, and H_2_O_2_ radicals with IC_50_ of 19.6 µg/mL, 56.5 µg/mL, and 60.5 µg/mL, respectively, as compared to ascorbic acids (IC_50_ of 10.7 µg/mL, and 10.2 µg/mL toward ABTS and DPPH radicals, respectively) and butylated hydroxytoluene (IC_50_ of 12.4 µg/mL toward H_2_O_2_ radicals) (Figure S29). Further, the examination of the metal scavenging activity and reducing power of CSE utilizing the CUPRAC, FRAP, and metal chelating assays revealed that CSE exhibits considerable reducing power and metal scavenging activity in a dose-dependent manner (Figure 2E,F). The evaluation of IC_50_ values indicated that CSE possesses IC_50_ of 126.4 µg/mL toward CUPRAC, 80.9 µg/mL toward FRAP, and 111.8 µg/mL toward metal chelating, as compared to ascorbic acid (IC50 of 19.8 µg/mL toward FRAP) and EDTA (IC_50_ of 232.3 µg/mL, and 20.9 µg/mL toward CUPRAC and metal chelating assays, respectively) (Figure S29). Together, these findings indicate that CSE exhibits a substantial antioxidant potential, as evidenced by its efficacy in scavenging the radicals but also its metal chelating power and reducing potential, which could be associated with the unique detected phytochemical profile of CSE determined by GC-MS, HPLC, and LC–MS analysis.

### 2.3. Pharmacological Network Analysis of CSE and PF Target Genes Identification

#### 2.3.1. Unique Gene Identification

**Figure 3:**
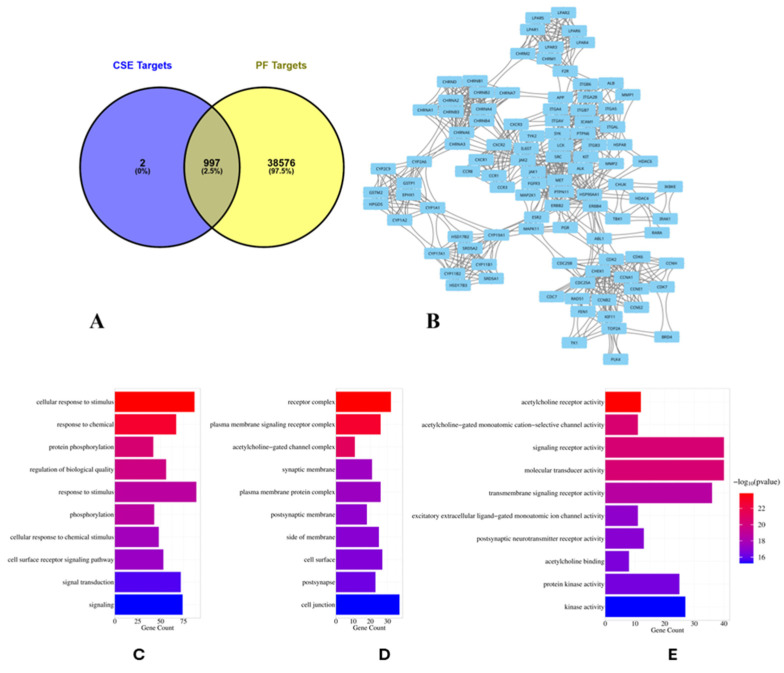
Integrated pharmacological network and functional enrichment analysis of CSE–PF targets. (**A**) Venn diagram showing the overlap of predicted CSE targets (999) with PF-associated genes (39,573). The intersection (997 genes, ~2.5% of PF targets) represents common targets, while 2 were unique to CSE and 38,576 were unique to PF. (**B**) Cluster 3 of the STRING–Cytoscape PPI network identified by MCODE, representing a densely connected module enriched in PF-related proteins. (**C**–**E**) Bar plots of the top 10 enriched Gene Ontology (GO) terms for Biological Process (BP), Cellular Component (CC), and Molecular Function (MF). The *x*-axis shows the number of associated genes, while the color gradient indicates statistical significance (*p*-values ranging from 2.35 × 10^−32^ to 2.60 × 10^−18^ for BP, 1 × 10^−9^ to 5 × 10^−10^ for CC, and 1.75 × 10^−15^ to 2.38 × 10^−11^ for MF).

To predict protein targets of CSE metabolites and assemble a robust PF gene set for intersection-based analysis, we applied a reverse-target (ligand-based) [39,40]: chemical structures were verified in PubChem (SMILES retrieved) and queried in SwissTargetPrediction, yielding 4307 putative targets across all compounds (Table S4). In parallel, PF-associated genes were aggregated from GeneCards, DisGeNET, and CTD, producing 276,411 database records in total (Table S5), with 8294 from GeneCards (Table S5), 30 from DisGeNET (Table S4), and 268,087 from CTD (Table S4). To derive nonredundant gene sets and quantify overlap between CSE-predicted targets and PF-associated genes, we removed duplicates and harmonized identifiers (Venny 2.1.0), which reduced the CSE list to 999 unique targets (Table S4) and the PF universe to 39,573 unique gene entries (Table S5). Intersection analysis identified 997 genes shared by CSE and PF (Figure 3A), indicating that virtually all CSE-predicted targets fall within the PF-associated gene space, yielding a focused set for downstream PPI hub and pathway enrichment analyses.

#### 2.3.2. PPI Network Analysis

To characterize the functional connectivity of the shared CSE–PF targets, we constructed a protein–protein interaction (PPI) network using the STRING database (confidence ≥ 0.700) and visualized it in Cytoscape. The resulting network contained 927 nodes and 12,588 edges (Table S6; Figure S30), representing predicted or validated physical and functional associations. Cluster analysis with the MCODE plugin identified 41 modules, among which cluster 3 stood out as the most prominent, comprising 103 nodes interconnected by 838 edges (Table S6; Figure 3B). These findings indicate that CSE–PF common targets form a highly connected network with distinct functional modules, suggesting the presence of key regulatory hubs that may mediate antifibrotic activity.

#### 2.3.3. Functional Enrichment Analysis

To explore the biological significance of the shared CSE–PF targets, cluster 3 of the PPI network was subjected to functional enrichment using g:Profiler. Analysis across the three GO categories identified extensive enrichment: 3550 biological processes (BP), 369 cellular components (CC), and 542 molecular functions (MF) (Table S7). To highlight the most relevant findings, the top 10–20 terms per category (adjusted *p* < 0.05) were selected and visualized (Figure 3C,E). BP enrichment (Figure 3C) revealed strong associations with PF-relevant processes, including cellular response to stimulus (GO:0051716), response to chemical exposure (GO:0042221), protein phosphorylation (GO:0006468), and regulation of biological quality (GO:0065008). These terms underscore the involvement of CSE–PF targets in signal transduction, stress responses, and post-translational regulation, all central to fibrotic remodeling. CC analysis (Figure 3D) highlighted enrichment in receptor complexes (GO:0043235), plasma membrane signaling receptor complexes (GO:0098802), and synaptic membranes (GO:0097060), pointing to roles in cell–cell communication, receptor signaling, and neurotransmission-like mechanisms that may influence inflammation and fibroblast activation. MF enrichment (Figure 3E) emphasized terms related to acetylcholine receptor activity (GO:0015464), transmembrane signaling receptors (GO:0004888), and molecular transducer activity (GO:0060089). These functions reflect a complex signaling environment in fibrotic lung tissue, where receptor-mediated processes contribute to immune modulation, epithelial–mesenchymal transition, and fibroblast activation. Together, these findings indicate that the CSE–PF shared targets converge on pathways related to stimulus response, receptor-mediated signaling, and phosphorylation-dependent regulation, supporting the hypothesis that CSE metabolites act through key fibrotic signaling networks.

#### 2.3.4. KEGG Pathway Analysis

**Figure 4:**
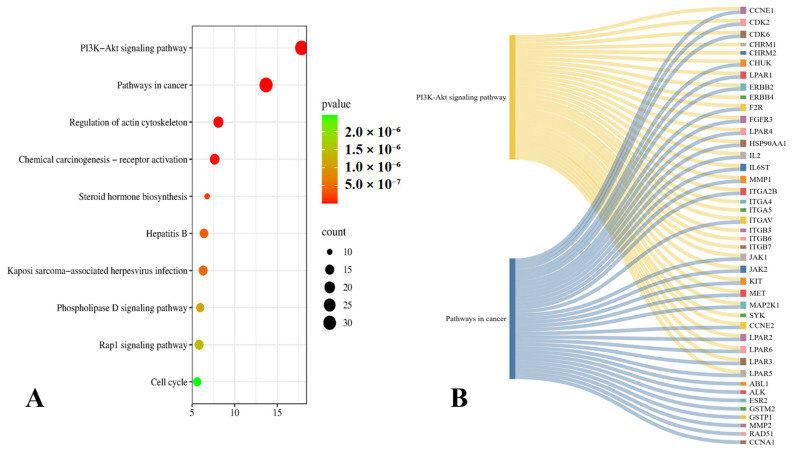
(**A**) Displaying the top 10 most significant enriched pathways generated by g:Profiler. The *X*-axis here represents the total genes involved in each of the pathways; meanwhile, the *Y*-axis represents the pathway names. The bubbles size is linked to the count value of the enriched genes, and the color gradient implies the *p*-value of the genes; the red color shows the vastly significant pathways with PI3K-Akt signaling pathway and pathways in cancers being the top ones. (**B**) The Sankey plot illustrates the Pathway-Gene network, where the left column represents the top enriched pathways, and the right column denotes the total number of genes associated with each pathway.

To further elucidate the molecular mechanisms underlying the effects of CSE in PF, KEGG pathway enrichment was performed using g:Profiler. A total of 171 significantly enriched pathways were identified (Table S7), with the top ten visualized in a bubble plot (Figure 4A). Among these, the PI3K–Akt signaling pathway and pathways in cancer emerged as particularly relevant to PF pathogenesis, consistent with their established roles in dysregulated cell survival, proliferation, and extracellular matrix (ECM) remodeling [41,42,43]. A Sankey diagram (Figure 4B) highlighted a core subset of overlapping genes, including CCNE1, CDK2, CDK6, CHUK, ERBB2, FGFR3, HSP90AA1, IL2, IL6ST, ITGA2B, ITGAV, JAK1, JAK2, KIT, MET, MMP1, MAP2K1, CCNE2, and LPAR1–6, that participate in both PI3K–Akt and cancer-related cascades. These recurrent genes likely function as key regulatory nodes within the fibrotic microenvironment. The enrichment of PI3K–Akt signaling underscores its role in fibroblast activation, epithelial–mesenchymal transition, and ECM accumulation, while the presence of cancer-related pathways reflects the shared features of fibrosis and malignancy, including uncontrolled proliferation, evasion of apoptosis, angiogenesis, and persistent tissue remodeling. Notably, several oncogenic drivers (e.g., ERBB2, JAK1/2, KIT, MET) were also implicated in PF-related processes such as fibroblast proliferation and cytokine signaling. Importantly, network pharmacology analysis showed that multiple CSE metabolites are predicted to target these shared components, including FGFR3, HSP90AA1, CDKs, and LPARs, suggesting a multi-target therapeutic potential. By converging on overlapping profibrotic and oncogenic pathways, CSE may exert broad-spectrum antifibrotic effects, attenuating fibroblast activation and restoring homeostatic signaling in lung tissue.

#### 2.3.5. Target Genes-miRNA Interaction Analysis

To investigate potential post-transcriptional regulation of key targets, we analyzed interactions between shared CSE–PF genes and microRNAs using the miRDB database. Given its established role in fibrosis, we focused on *hsa-miR-let-7d*, a member of the *let-7* family known for regulating oncogenic and fibrogenic signaling and reported to be downregulated in lung fibroblasts from PF patients [44,45,46]. Among the overlapping genes identified in the two most enriched KEGG pathways, IL6 and MMP1 were predicted as direct targets of *miR-let-7d*, with interaction scores of 53 and 51, respectively (Figures S31 and S32). Both genes play central roles in inflammation, ECM remodeling, and fibrotic progression. These findings suggest that *miR-let-7d* may exert antifibrotic effects by repressing IL6- and MMP1-mediated signaling, highlighting a potential regulatory axis through which CSE metabolites could influence fibrosis.

#### 2.3.6. miRNA-lncRNA Interaction Analysis

To explore whether *miR-let-7d* might be functionally regulated by long non-coding RNAs (lncRNAs), we performed an in silico analysis using the DIANA-lncBase v3.0 platform. The analysis predicted several lncRNAs with strong binding potential to *hsa-let-7d-5p*, all with miTG scores > 0.99, indicating high-confidence interactions (Table S8). Among these, KCNQ1OT1 (ENSG00000269821; score = 0.992) is well known for roles in chromatin remodeling and gene silencing [47], while TPTEP1 (ENSG00000100181; score = 0.998) has been linked to regulation of cell growth and tumorigenesis. In addition, three computationally defined loci (XLOC_013024, XLOC_008765, XLOC_013931) were identified. Although not yet experimentally characterized, such XLOC transcripts may act as competitive endogenous RNAs (ceRNAs), sequestering *miR-let-7d*. These findings suggest that specific lncRNAs, including both established regulators and novel XLOC candidates, could form part of a lncRNA–miRNA–mRNA regulatory axis in PF, and further highlight the impact of CSE on the modulation of this modulatory axis.

### 2.4. In Vivo Validation of the Antifibrotic Potential of CSE in an Mtx-Induced PF Model

#### 2.4.1. CSE Elevates *miR-let-7d* Expression by Suppressing *ncNRFR* in Fibrotic Lung Tissue

**Figure 5:**
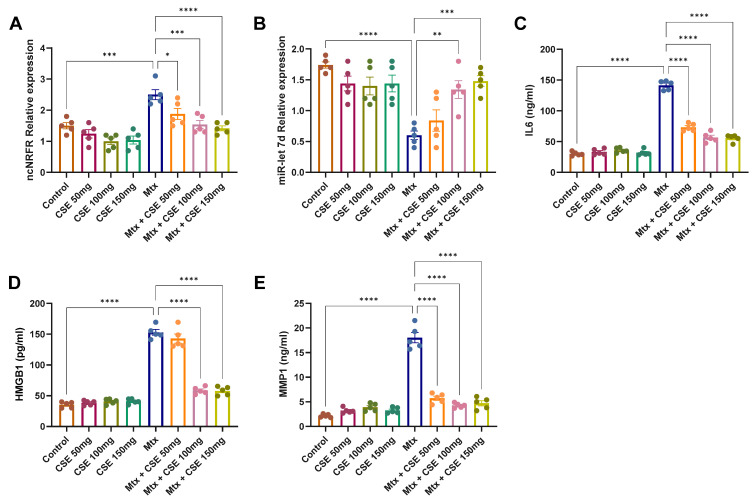
Dose-dependent effects of CSE on *ncNRFR/miR-let-7d* axis and inflammatory–fibrotic markers in Mtx-induced PF. (**A**,**B**) *ncNRFR* and *miR-let-7d* expression levels. (**C**–**E**) IL6, HMGB1, and MMP1 levels across groups. Data represent mean ± SE (n = 5). Significance: **** *p* ≤ 0.0001, *** *p* ≤ 0.001, ** *p* ≤ 0.01, * *p* ≤ 0.05.

To experimentally validate the predicted lncRNA–miRNA interactions, we assessed the expression of *ncNRFR* and *miR-let-7d* in an Mtx-induced pulmonary fibrosis model. Although *ncNRFR* was not among the top-ranked candidates in the purely score-based computational predictions, it was selected for experimental validation based on its previously documented role in regulating the *let-7* microRNA family and its known involvement in epithelial injury and stress-related signaling pathways. Because *let-7d* dysregulation is central to fibrogenesis and epithelial repair, *ncNRFR* represented a biologically compelling candidate despite its intermediate computational ranking. *ncNRFR* is a long non-coding RNA reported to suppress members of the *let-7* family, thereby influencing fibrogenic signaling [48,49]. qRT-PCR analysis revealed a marked induction of *ncNRFR* in the Mtx-treated group compared with controls (*p* < 0.001), confirming successful fibrosis establishment (Figure 5). Treatment of healthy controls with CSE (50–150 mg/kg) did not significantly alter *ncNRFR* levels, indicating no effect under basal conditions. In contrast, CSE administration to Mtx-treated animals significantly attenuated *ncNRFR* expression in a dose-dependent manner, with the most pronounced reduction observed at 150 mg/kg. Given the inverse relationship between *ncNRFR* and *let-7* miRNAs, we next examined *miR-let-7d* expression. As expected, Mtx-treated lungs showed a robust suppression of *miR-let-7d* compared with controls (*p* < 0.0001) (Figure 5A,B). CSE treatment alone had no effect on *miR-let-7d* in healthy lungs. However, in the Mtx group, CSE supplementation restored *miR-let-7d* expression in a dose-dependent fashion, with significant recovery at 150 mg/kg, while lower doses showed a positive but less pronounced trend. Together, these results suggest that CSE exerts antifibrotic effects in vivo by downregulating *ncNRFR* and reinstating *miR-let-7d* expression in lung tissue, consistent with the regulatory axis predicted from our bioinformatics analysis. Our findings indicate that *ncNRFR* and *let-7d* are both significantly modulated in MTX-induced pulmonary fibrosis and partially normalized by CSE, suggesting a potential regulatory association rather than a direct mechanistic interaction.

#### 2.4.2. CSE Ameliorates Inflammatory (IL6, HMGB1) and Fibrotic (MMP1) Targets of *miR-let-7d* in Mtx-Induced PF

Following the bioinformatics prediction of IL6 and MMP1 as downstream targets of *miR-let-7d*, we next investigated their biological relevance in vivo, together with HMGB1, a critical regulator downstream of IL6. ELISA analyses confirmed that Mtx administration strongly induced all three markers, with IL6 increased 4.7-fold, HMGB1 increased 4.4-fold, and MMP1 increased 8.3-fold compared to controls (*p* < 0.0001 for all) (Figure 5). These results validate that the Mtx model successfully recapitulates the inflammatory and fibrotic milieu characteristic of PF. When administered alone to control animals, CSE at doses of 50–150 mg/kg did not significantly alter the expression of IL6, HMGB1, or MMP1, suggesting that the extract exerts no basal effect on lung tissue homeostasis. However, CSE supplementation in Mtx-induced PF animals markedly reduced the elevated levels of these markers in a dose-dependent manner, with the most pronounced effects observed at 150 mg/kg, where levels approached those of the control group (Figure 5). Together, these findings demonstrate that CSE effectively attenuates pro-inflammatory (IL6, HMGB1) and pro-fibrotic (MMP1) signaling in vivo. By targeting this axis, which is tightly connected to the *miR-let-7d* regulatory network, CSE not only restores balance in inflammatory and fibrotic pathways but also supports the mechanistic predictions derived from our network pharmacology analysis.

#### 2.4.3. CSE Modulates TGF-β and E-Cadherin Expression in Mtx-Induced PF

**Figure 6:**
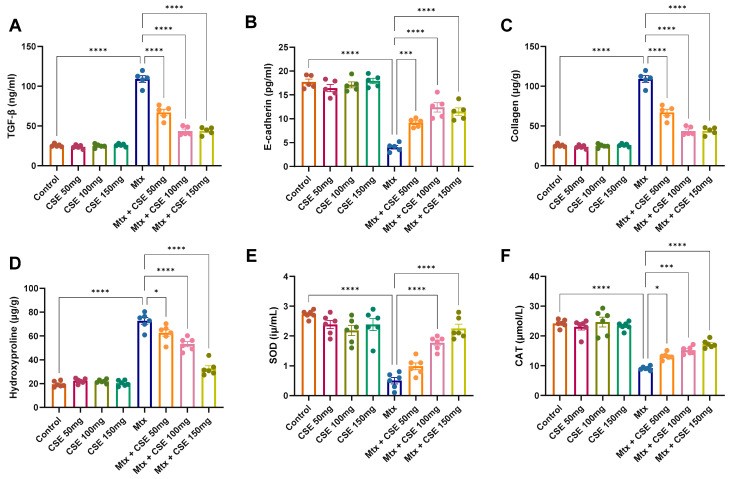
Dose-dependent effects of CSE on profibrotic and antioxidant biomarkers in the Mtx-induced pulmonary fibrosis model. (**A**,**B**) TGF-β and E-cadherin expression. (**C**,**D**) Collagen and hydroxyproline levels. (**E**,**F**) Superoxide dismutase (SOD) and catalase (CAT) activities. Data are presented as mean ± SE (n = 5). Significance is shown relative to the Mtx group (**** *p* ≤ 0.0001, *** *p* ≤ 0.001, * *p* ≤ 0.05).

Epithelial–mesenchymal transition (EMT) is a hallmark of pulmonary fibrosis, characterized by the loss of E-cadherin and excessive activation of TGF-β–driven fibrotic signaling [50,51]. To assess whether CSE influences this axis, we quantified TGF-β and E-cadherin expression levels in the different experimental groups using ELISA. As shown in Figure 6, the Mtx-induced PF model displayed a pronounced increase in TGF-β expression (*p* < 0.0001) and a marked reduction in E-cadherin levels (*p* < 0.0001) compared with untreated controls. These changes are consistent with EMT activation and confirm the establishment of fibrosis. Administration of CSE to healthy controls (50–150 mg/kg) produced no significant alterations in either TGF-β or E-cadherin, further supporting that the extract has no baseline effect on normal lung tissue. In contrast, CSE treatment of Mtx-induced PF animals significantly and dose-dependently modulated these markers. Beginning at doses >50 mg/kg, CSE reduced TGF-β levels toward those of the control group, while simultaneously restoring E-cadherin expression, with highly significant increases at 100 and 150 mg/kg (*p* < 0.0001). These findings support that CSE counteracts Mtx-induced EMT by suppressing TGF-β and restoring epithelial integrity through E-cadherin upregulation. In line with the observed downregulation of IL6, HMGB1, and MMP1, this suggests that CSE interrupts interconnected inflammatory and fibrotic signaling networks, thereby protecting against pathological lung remodeling.

#### 2.4.4. CSE Reduces Collagen and Hydroxyproline Accumulation in Mtx-Induced PF Lung Tissue

Given the observed modulation of profibrotic mediators such as TGF-β and E-cadherin, we next evaluated whether CSE could also influence the structural hallmarks of fibrosis by assessing collagen deposition and hydroxyproline content, two well-established markers of fibrotic remodeling [52,53,54,55]. As expected, the Mtx-treated PF model exhibited a marked increase in both collagen and hydroxyproline levels compared with untreated controls (*p* < 0.0001), confirming robust induction of fibrotic pathways (Figure 6). Administration of CSE alone (50–150 mg/kg) did not significantly alter these markers in control animals, suggesting no basal effect on normal lung extracellular matrix turnover. In contrast, CSE treatment of Mtx-induced PF animals significantly and dose-dependently attenuated the elevated collagen and hydroxyproline levels. The reductions were most pronounced at 100 and 150 mg/kg, where values approached those observed in the control group. These findings reveal that CSE not only modulates molecular regulators of fibrosis but also limits the downstream structural deposition of collagen and accumulation of hydroxyproline, thereby directly reducing fibrotic remodeling in lung tissue.

#### 2.4.5. CSE Restores Serum Antioxidant Enzyme Activity (SOD and Catalase) in Mtx-Induced PF

To evaluate whether CSE modulates antioxidant defenses, we measured serum SOD and catalase activity across the experimental groups. As shown in Figure 6, Mtx administration significantly decreased both SOD and catalase levels compared with untreated controls (*p* < 0.0001), confirming induction of oxidative stress in the PF model. In contrast, CSE administration to healthy control animals did not significantly alter antioxidant enzyme levels, indicating no baseline effect on redox homeostasis. Importantly, CSE supplementation in Mtx-induced PF animals reversed the Mtx-mediated suppression of SOD and catalase in a dose-dependent manner. At 50 mg/kg, CSE produced a modest but detectable increase, with a significant improvement in catalase (*p* < 0.05) and a positive, though not statistically significant, trend for SOD (*p* = 0.2098). At 100 mg/kg, CSE treatment yielded a highly significant increase in both SOD and catalase (*p* < 0.001). The effect was most pronounced at 150 mg/kg, where both enzymes were restored to near-control levels (*p* < 0.0001) (Figure 6E,F). These in vivo findings complement our in vitro antioxidant assays, indicating that CSE not only exerts direct radical-scavenging and metal-chelating activity but also enhances endogenous enzymatic defenses (SOD and catalase). Together, these dual antioxidant mechanisms are likely to contribute to the overall antifibrotic effect of CSE in pulmonary fibrosis.

#### 2.4.6. CSE Attenuates Fibrous Tissue Deposition and Interalveolar Septal Thickness and Restores Lung Parenchyma Architecture in Mtx-Induced PF

**Figure 7:**
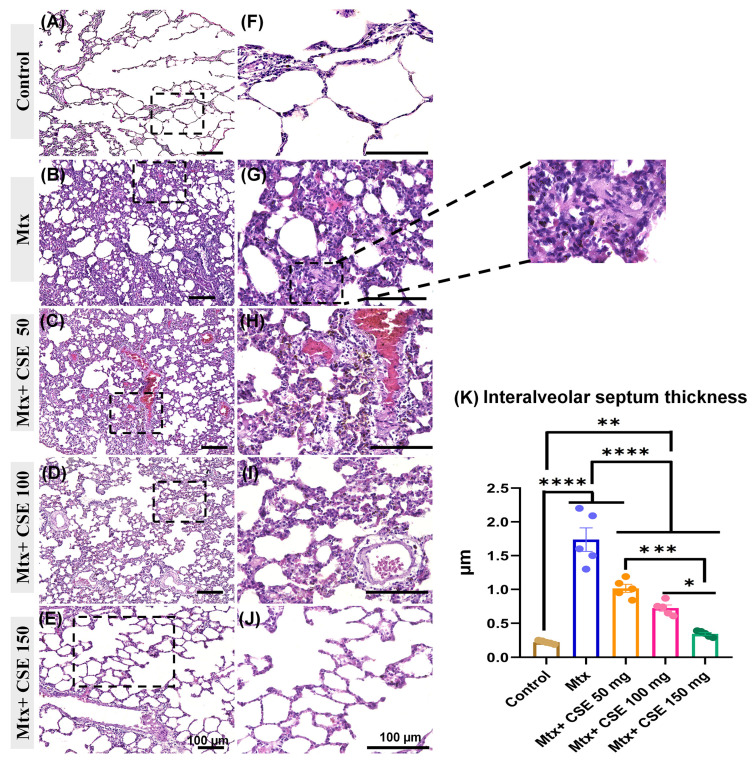
Histopathological and morphometrical analysis of HE-stained lung tissues among experimental models. (**A**–**J**) Histopathological features of HE-stained lung sections of the control group (**A**), model (Mtx induced fibrosis) group (**B**,**G**), Mtx+ CSE 50 group (**C**), Mtx+ CSE 100 group (**D**), and Mtx+ CSE 150 group (**E**). (**F**–**J**) Higher magnification of the boxed area in (**A**), (**B**), (**C**), (**D**), (**E**), respectively. Notice normal architecture of lung tissue with thin interalveolar septa in both control and Mtx+ CSE 150 group (**A**,**E**,**F**,**J**). More collagen fiber deposition, alveolar distortion, congested blood vessels, and thick interalveolar septum in model group (**B**,**G**). Moderate to mild collagen deposition, and congested blood vessels in Mtx+ CSE 50 (**C**,**H**), and Mtx+ CSE 100 (**D**,**I**) groups, respectively. (**K**) Graph showing the interalveolar septum thickness among studied groups. The data are shown for the experimental groups with n = 5 (mean± SE) and considered as significant at *p* ≤ 0.05 (**** *p* ≤ 0.0001, *** *p* ≤ 0.001, ** *p* ≤ 0.01, and * *p* ≤ 0.05).

Finally, we aimed to further affirm the anti-fibrotic potential of CSE in mitigating the progression of lung fibrosis in Mtx-induced PF by conducting detailed histological and immunohistochemical assessments of the lung tissues across the control, Mtx-treated, Mtx + CSE 50mg/kg, Mtx + CSE 100 mg/kg, and Mtx + CSE 150 mg/kg groups. Analysis of the lung sections stained with HE from the control group revealed a normal lung architecture consisting of a normal alveolar structure with a thin interalveolar septum and a normal bronchial and vascular structure (Figure 7A,F). The lung tissues of the Mtx-treated group showed distorted lung parenchyma with advanced areas of fibrosis alternating with non fibrotic lung parenchyma, and thick interalveolar septa. Several collapsed alveoli with numerous congested blood vessels were also observed (Figure 7B,G). On the other hand, the CSE-treated groups showed varied and dose-dependent degrees of improvement in the lung lesions. In this regard, the CSE (50 mg/kg)-treated group (Mtx + CSE50) revealed less improvement, with a patchy appearance of attenuated collagen deposition, numerous distorted alveoli with a thick interalveolar septum, and severely congested blood vessels (Figure 7C,H). Milder restoration was observed in the CSE (100 mg/kg)-treated group (Mtx + CSE100) with more restoration of lung architectures, and less congested blood vessels in the areas of the honeycomb (Figure 7D,H). Interestingly, the CSE (150 mg/kg)-treated group (Mtx + CSE150) exhibited significant improvements with normal alveolar shape and thin interalveolar septa and blood vessels (Figure 7E,I). The morphometric measurements of the interalveolar septum revealed significantly (*p* ≤ 0.0001) higher thickness in the Mtx-treated group as compared to the control and CSE-treated groups. Furthermore, compared to the control groups, significantly higher thickness was reported for the Mtx + CSE50 and Mtx + CSE100 but not Mtx + CSE150 (Figure 7K). On the other hand, the CSE treatment demonstrated a considerable (*p* ≤ 0.0001) and dose-dependent reduction in the thickness as compared to the Mxt-model. In agreement with our biochemical analysis, these results support the antifibrotic potential of CSE in lung fibrosis, with a more pronounced effect at higher doses.

#### 2.4.7. CSE Diminishes Collagen Fiber Deposition in the Lung Parenchyma of Mtx-Induced PF

**Figure 8:**
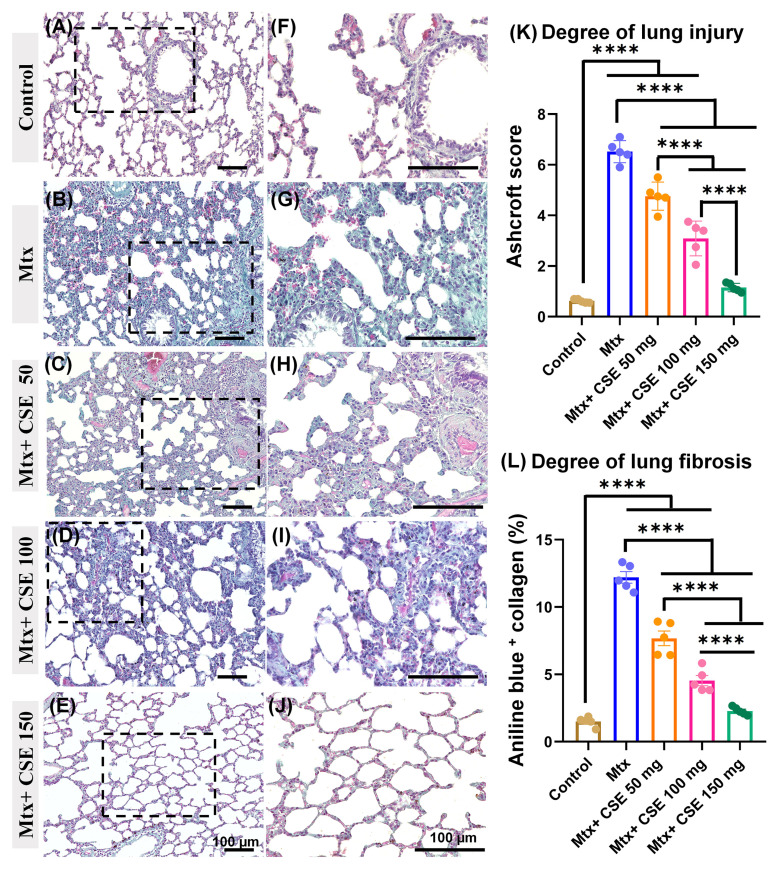
Histopathological features and morphometrical analysis of indices of lung tissue among studied groups. (**A**–**J**) Histopathological features of Masson’s trichrome-stained lung sections of the control group (**A**), model (Mtx induced fibrosis) group (**B**,**G**), Mtx+ CSE 50 group (**C**), Mtx+ CSE 100 group (**D**), and Mtx+ CSE 150 group (**E**). (**F**–**J**) Higher magnification of the boxed area in (**A**), (**B**), (**C**), (**D**) and (**E**), respectively. Notice normal architecture of lung tissue with scarce peribronchial and interalveolar aniline blue positive collagen fiber deposition in both control and Mtx+ CSE 150 group (**A**,**E**,**F**,**J**), apparently numerous aniline blue positive collagen fiber deposition in model group (**B**,**G**). Moderate, and mild collagen deposition congested blood vessels in Mtx+ CSE 50 (**C**,**H**), and Mtx+ CSE 100 (**D**,**I**) groups, respectively. (**K**,**L**) Graphs showing the degree of lung injury among studied groups indicated by Ashcroft score (**K**), and percentage of aniline blue + collagen fibers (**L**). The data are shown for the experimental groups with n = 5 (mean ± SE) and considered as significant at *p* ≤ 0.05 (**** *p* ≤ 0.0001).

To further assess the degree of fibrosis in lung tissues across the studied groups, we examined the lung sections stained with MT. As shown in (Figure 8A,F), the control group showed scarce collagen fiber deposition in the peribronchial and lung parenchyma. Conversely, the Mtx-treated group exhibited extensive collagen deposition represented by extensive positive aniline blue staining in both lung parenchyma and peribronchial spaces with numerous congested blood vessels (Figure 8B,G). The lung sections of the Mtx+ CSE50 (Figure 8C,H) and Mtx + CSE100 (Figure 8D,H) groups revealed mild to moderate collagen fiber deposition. Interestingly, the Mtx + CSE150 group showed minor collagen fiber deposition in the parenchyma with normal blood vessels (Figure 8E,I). To further quantify the damage to lung tissues, the Ashcroft score was applied. The Mtx-treated PF model showed an extensively (*p* ≤ 0.0001) higher score as compared to the control and CSE-treated groups. Furthermore, the Mtx + CSE150 group displayed (*p* ≤ 0.0001) more than a 3-fold reduction in Ashcroft score as compared to the Mtx-treated model, with no significant differences as compared to the control group. However, both Mtx + CSE50 and Mtx + CSE100 groups revealed significantly (*p* ≤ 0.0001) higher scores as compared to the control and Mtx + CSE150 groups (Figure 8K). Furthermore, to investigate the degree of lung fibrosis, the averages of the aniline blue + areas were compared across the studied groups. Similarly to the Ashcroft score, a considerably (*p* ≤ 0.0001) higher percentage of collagen fibers with aniline blue + was demonstrated in the Mtx-treated group as compared to other studied groups. Additionally, the Mtx + CSE150 group displayed a significant (*p* ≤ 0.0001) reduction in this percentage as compared to the Mtx-treated model, Mtx + CSE50, and Mtx + CSE100 groups, with no significant difference from the control group (Figure 8L). Our findings, aligned with the hematoxylin and eosin (H&E) results, underscore the antifibrotic potential of CSE, especially at high doses, to mitigate the deterioration in lung tissues associated with Mtx treatment.

#### 2.4.8. CSE Reduces the Positive Staining for the α-SMA in the Lung Parenchyma of Mtx-Induced PF

**Figure 9:**
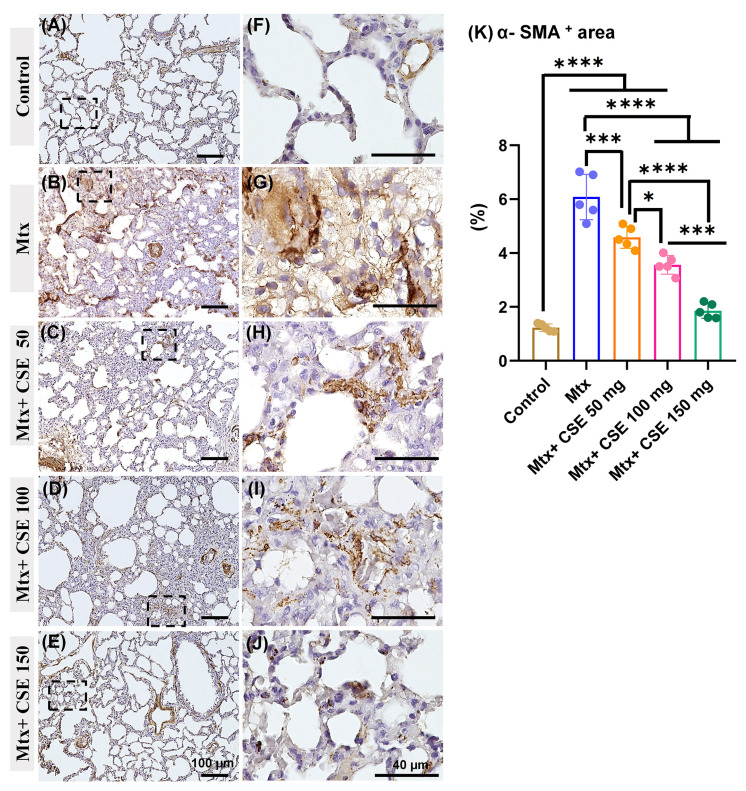
Immunohistochemical and morphometrical analysis of the index of lung tissue among the studied groups. (**A**–**J**) α-SMA- immunohistochemical-stained lung sections of the control group (**A**), model (Mtx induced fibrosis) group (**B**,**G**), Mtx+ CSE 50 group (**C**), Mtx+ CSE 100 group (**D**), and Mtx+ CSE 150 group (**E**). (**F**–**J**) Higher magnification of the boxed area in (**A**), (**B**), (**C**), (**D**), (**E**), respectively. Notice less α-SMA positive staining around the blood vessel and within the lung parenchyma in both the control and Mtx + CSE150 group (**A**,**E**,**F**,**J**), and apparently extensive α-SMA positive staining in model group (**B**,**G**). Moderate, and mild α-SMA positive staining in Mtx+ CSE 50 (**C**,**H**), and Mtx+ CSE 100 (**D**,**I**) groups, respectively. (**K**) Graph showing the percentage of α-SMA positive staining among the studied groups. The data are shown for the experimental groups with n = 5 (mean ± SE) and are considered significant at *p* ≤ 0.05 (**** *p* ≤ 0.0001, *** *p* ≤ 0.001, and * *p* ≤ 0.05).

To gain further insights into the degree of lung damage and fibrosis, we assessed α-SMA-stained lung tissues across the studied groups. Our analysis revealed a little positive staining for the α-SMA around the blood vessel wall with apparently scarce positive staining in the lung parenchyma of the control group (Figure 9A,F) and Mtx + CSE150 (Figure 9E,I). However, extensive positive staining was observed in the lung parenchyma as well as around the blood vessels of the Mtx-treated model (Figure 9B,G). The lung sections of the Mtx + CSE50 (Figure 9C,H) and Mtx + CSE100 (Figure 9D,H) groups revealed less positive staining in the lung parenchyma and around blood vessels, as compared to the Mtx-treated group, while more apparently as compared to the control model. The Mtx + CSE150 model (Figure 9E,I), similar to the control model, displayed limited positive staining for the α-SMA in the lung parenchyma and around the blood vessel wall. Further, morphometric measurement of the percentages of α-SMA positive area in the lung sections of the model group revealed a significantly higher percentage as compared to the control model (*p* ≤ 0.0001) and other studied groups (*p* ≤ 0.0001, and *p* ≤ 0.001). Interestingly, the Mtx + CSE150 group showed a significant (*p* ≤ 0.0001) reduction in α-SMA percentage as compared to the Mtx-treated model, Mtx + CSE50, and Mtx + CSE100 groups, with no significant differences from the control group (Figure 9K.

## 3. Discussion

Pulmonary fibrosis is a progressive disorder characterized by aberrant epithelial repair, chronic inflammation, oxidative stress, and excessive extracellular matrix (ECM) deposition. These interconnected mechanisms ultimately result in irreversible tissue remodeling and functional decline. Identifying new antifibrotic candidates requires not only demonstrating biological activity but also understanding how bioactive metabolites interact with fibrosis-related pathways. In this study, we used a multi-tiered in vitro, in silico, and in vivo approach to characterize the biological activity of *CSE* within this mechanistic framework. CSE showed dose-dependent antiproliferative activity against malignant lung-derived cell lines (HEp-2 and A549), while demonstrating lower toxicity toward WI-38 normal fibroblasts. Although this pattern suggests differential responsiveness, the selectivity indices (SI 1.8–2.66) fall below accepted thresholds for meaningful selectivity, indicating the need for future studies using primary epithelial cells and extended dose–response evaluation. These findings align with previous work documenting cytotoxic effects of CSE against HepG2, PC-3, and HuH-7 cells [56,57,58]. CSE also exhibited substantial antioxidant potential across ABTS, DPPH, and H_2_O_2_ assays, with IC_50_ values significantly lower than earlier reports [59,60,61]. Such differences likely reflect variations in extraction methods, plant genotype, or environmental factors known to influence phytochemical composition. CSE’s metal-reducing and chelating capacity, demonstrated through CUPRAC, FRAP, and ferrozine assays, is consistent with prior observations of its redox-active properties [29,30,62].

**Figure 10:**
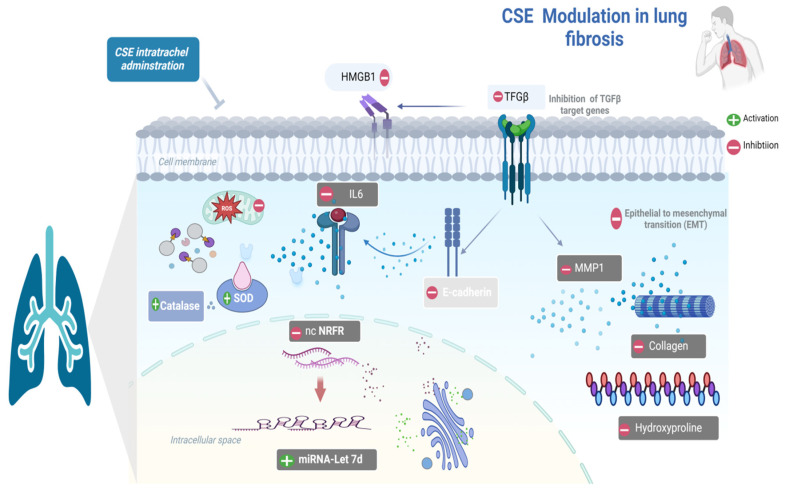
Summarization of the different modes of action for CSE in the modulation of lung fibrosis.

Network pharmacology highlighted 997 protein targets shared between CSE metabolites and pulmonary fibrosis–associated genes, with enrichment in pathways related to inflammation, oxidative stress, and protein phosphorylation. IL6 and MMP1 emerged as central hubs, aligning with their recognized role in driving inflammatory cascades, fibroblast activation, and ECM remodeling. The analysis also identified *miR-let-7d* and suggested that *ncNRFR* could interact with *let-7* family members based on reported biological relationships. Although ncNRFR was not the top-ranked interaction candidate computationally, its established involvement in *let-7* processing and epithelial stress responses provided the rationale for prioritizing its experimental evaluation. To substantiate the molecular mechanisms underlying the antifibrotic activity of CSE, we combined in silico pharmacological network analysis with in vivo validation in the Mtx-induced pulmonary fibrosis model. The MTX-induced PF model was selected for in vivo investigations due to its clinical relevance and because it effectively reproduces oxidative imbalance, inflammatory activation, and epithelial injury processes central to fibrosis pathogenesis [36,37,38]. In fibrotic lungs, ncNRFR was upregulated while *miR-let-7d* was downregulated, consistent with prior reports linking non-coding RNA dysregulation with pulmonary fibrosis [44,63,64,65]. Treatment with CSE reversed these changes in a dose-dependent manner, thereby restoring the putative expression levels of *ncNRFR* and *miR-let-7d*. While our data provide clear evidence that CSE modulates *ncNRFR* and let-7d expression in vivo, these findings remain correlative. The present study does not establish a direct physical interaction between *ncNRFR* and let-7d, nor does it demonstrate that modulation of this relationship is required for the antifibrotic effects of CSE. Our study represents an important direction for future work to explore the definitive mechanistic confirmation by luciferase reporter assays. Similarly, CSE significantly reduced IL6 and HMGB1, key mediators elevated during inflammation-driven fibrosis [66,67,68,69], and improved ECM remodeling markers, including collagen, hydroxyproline, and MMP1 [70,71,72]. Histological evaluation demonstrated that CSE mitigated collagen fiber accumulation and restored lung architecture. CSE also normalized EMT-related alterations, suppressing TGF-β and α-SMA while increasing E-cadherin expression [50,73,74]. Moreover, reductions in SOD and catalase activity observed in MTX-treated rats were restored following CSE supplementation, underscoring its antioxidant activity [75,76,77,78]. Collectively, these findings indicate that CSE modulates multiple processes implicated in fibrosis progression, although precise mechanistic pathways remain to be elucidated (Figure 10). An apparent disparity arises between the potent antiproliferative activity of CSE in vitro and its cytoprotective, antifibrotic effects in vivo. This can be explained by differences in cellular context and exposure conditions. The in vitro experiments were performed on malignant cell lines (A549 and Hep2), which possess high proliferative rates, altered redox homeostasis, and heightened sensitivity to apoptosis-inducing stimuli. CSE showed lower toxicity toward normal lung fibroblasts (Wi38) and did not induce lung injury in control rats receiving CSE alone, supporting a degree of selectivity toward malignant rather than non-malignant lung cells. Additionally, in vivo exposure reflects oral dosing and systemic metabolism, which are not directly comparable to fixed in vitro concentrations. Given that pulmonary fibrosis carries an increased risk of lung carcinogenesis, the selective antiproliferative activity observed against the lung adenocarcinoma A549 line may represent a potentially beneficial property of CSE. Nonetheless, further studies using primary lung epithelial cells and models of fibrosis-associated carcinogenesis are needed to refine our understanding of this dual activity.

A comprehensive metabolite profile generated through GC–MS, HPLC, and UPLC–T-TOF–MS/MS revealed that CSE contains a chemically diverse mixture of metabolites, including terpenoids, flavonoids, phenolics, glucosinolates, amino acid derivatives, vitamins, nucleosides, and oligosaccharides. GC–MS analysis showed a predominance of terpenoids, followed by fatty acids, hydrocarbons, alkaloids, and sterols, consistent with earlier investigations of CSE composition [29,30,59,60,79,80]. Targeted HPLC identified substantial quantities of catechol, syringic acid, rutin, quercetin, vitamin B2, and vitamin K, while untargeted UPLC–MS extended coverage to 105 metabolites across glucosinolates, hydroxycinnamic acids, amino acid derivatives, flavonols, and carbohydrates [62,81,82,83].

The alignment between these dominant metabolite classes and the biological responses observed in vivo suggests that several high-abundance phytochemicals likely contribute to CSE’s antioxidant, anti-inflammatory, and antifibrotic activity. Phenolic and flavonoid compounds such as quercetin, rutin, catechol, syringic acid, and related hydroxycinnamate derivatives, present at quantifiable levels, are widely recognized for reducing oxidative stress and attenuating cytokine signaling [84,85,86], consistent with the restoration of SOD and catalase and decreased IL6 and HMGB1 in CSE-treated animals. Glucosinolate derivatives, particularly benzyl glucosinolate detected in UPLC–MS analysis, give rise to benzyl isothiocyanate (BITC), which has been shown to inhibit COX/LOX signaling, T-cell activation, IL6 production, and collagen deposition [87,88,89], aligning with CSE’s impact on inflammatory and ECM biomarkers. Amino acid derivatives such as N,N-dimethylglycine (DMG) and tryptophan metabolites exhibit ROS-scavenging and cytokine-modulating effects [90,91,92], supporting improvements in oxidative balance and inflammatory mediators. Additionally, phenylpropanoids including rosmarinic acid–related constituents and sinapic acid derivatives have documented antifibrotic effects via modulation of TGF-β1/ROS and EMT pathways [93,94,95], paralleling the reductions in α-SMA, TGF-β, and collagen observed here. Terpenoids identified in the GC–MS profile, including γ-terpineol, cis-4-thujanol, n-hexadecanoic acid, and caryophyllene, possess antioxidant or cytotoxic properties that may contribute to CSE’s radical-scavenging activity and differential cytotoxicity [96,97,98,99,100,101]. Finally, carbohydrate metabolites such as sucrose and melibiose, known to attenuate TNF-α–mediated inflammation [102,103,104,105], correspond with reductions in inflammatory mediators in vivo. Together, these data indicate that CSE’s biological activity is plausibly derived from the combined actions of multiple metabolite classes rather than from a single dominant compound.

The convergence of phytochemical diversity, network predictions, and in vivo outcomes supports a model in which CSE modulates several interconnected processes central to fibrosis progression, including oxidative stress, cytokine signaling, fibroblast activation, EMT, and ECM remodeling. These broad effects likely reflect metabolite synergy, a characteristic feature of complex plant extracts. Modulation of *ncNRFR* and *let-7d* expression may represent an additional regulatory layer influencing epithelial homeostasis; however, this remains associative and requires direct mechanistic validation. Several limitations should be considered: the Mtx model reflects drug-induced rather than idiopathic pulmonary fibrosis, only male Wistar rats and a single dosing regimen were used, and the modest sample size may not capture smaller effects. These factors, along with potential species-specific differences in phytochemical metabolism and ncRNA regulation, limit direct extrapolation of the findings to human disease. Nevertheless, the conserved involvement of oxidative stress, cytokine networks, ECM remodeling, and EMT across mammalian fibrosis models suggests potential translational relevance. Further work in additional fibrosis models, primary human lung cells, and ncRNA-targeted mechanistic systems is required to define the causal contribution of the *ncNRFR/let-7d* axis and the clinical potential of CSE or its active metabolites.

## 4. Materials and Methods

### 4.1. Drugs and Chemicals

Methotrexate (Mtx) was procured from El-Gomhouria Company (Cairo, Egypt). The solvents were acquired in high grade from Sigma-Aldrich (Merck KGaA, Darmstadt, Germany) and Fischer Scientific (Loughborough, UK).

### 4.2. Plant Collection and Extraction

CS was obtained from the Agricultural Research Center (Ministry of Agriculture in Cairo, Egypt) and was identified by Maha Elshamy (Assistant Professor) at Mansoura University, Department of Botany. Samples were preserved in the Herbarium of Mansoura University (HMU: 36575). Following identification, seeds were washed to remove impurities and then dried in an air-ventilated oven at temperatures of 40–60 °C. Once dried, seeds were ground into a powder and passed through an 80-mesh screen for uniformity. For extraction, 500 g of the sample was soaked in 1 L of 99% methanol for three days at room temperature. The mixture was filtered using Whatman grade-1 filter paper in a vacuum funnel, followed by rotary evaporation (RE200, Bibby Sterling, Ltd., Staffordshire, UK) at 4 rpm and 65 °C to remove the solvent and obtain a concentrated extract. The crude methanolic extract was lyophilized to obtain a dry powder, yielding 45 g (9% of the original weight). Samples were stored at 4 °C until further analysis [106].

### 4.3. Chemical Characterization

#### 4.3.1. HPLC Analysis

A comprehensive phytochemical screening was performed to quantify phenolic and flavonoid constituents in the CSE using an Agilent 1100 HPLC system (Agilent Technologies, Santa Clara, CA, USA) equipped with dual capillary LC pumps, an autosampler, a solvent degassing unit, and a 2489 UV/Vis detector. Flavonoids were monitored at 360 nm, while phenolic acids were monitored at 250 nm. For flavonoid separation, the mobile phase consisted of acetonitrile (Solvent A) and 0.2% (*v*/*v*) aqueous formic acid (Solvent B) under isocratic elution (70:30, *v*/*v*) at a flow rate of 1 mL/min. The injection volume was 25 µL, and the column temperature was maintained at 25 °C. For phenolic acids, a gradient system of methanol (Solvent A) and acetic acid (Solvent B) was used: 0–3 min, 100% B; 3–8 min, 50% A; 8–10 min, 80% A; 10–15 min, 50% A. The injection volume was 20 µL. Quantification was performed using external standard calibration. Authentic standards of catechol, syringic acid, cinnamic acid, caffeic acid, salicylic acid, ferulic acid, quercetin, rutin, luteolin, apigenin, naringin, and kaempferol were prepared at five concentrations (1–50 µg/mL). Calibration curves were generated by plotting peak area versus concentration, yielding linearity values of R^2^ ≥ 0.998 for all standards. The CSE sample was prepared at 10 mg/mL in methanol, filtered, and injected under identical chromatographic conditions. Individual compound concentrations were calculated by interpolating the sample peak areas into the respective standard calibration curves and expressed as µg/mL of the injected extract solution, a commonly used quantification unit in phytochemical HPLC profiling. Identification of analytes was confirmed by matching retention times and UV spectra with their respective standards [107].

#### 4.3.2. Chromatography-Mass Spectrometry (GC-MS) Phytochemical Profiling

10 g of the powdered CSE were homogenized, then they were soaked in one hundred milliliters of 85% methanol in a sealed container and left to stand for twenty-four hours at room temperature. Following this initial maceration, the mixture underwent conventional extraction through sonication at 40 °C for 60 min. Subsequently, the CSE was filtered and condensed under a rotovap vacuum at 40 °C. CSE was then methylated and prepared for GC-MS analysis by adding H_2_SO_4_ -methanol 2% (*v*/*v*) to10 mg CSE and stirring at 80 °C, then mixed with 1 mL of 0.8 M KCl and 0.25 mL of sodium hydroxide and shaken. After adding 1 mL of hexane, the top layer was removed for GCMS analysis [107]. The methanolic CSE samples were analyzed using a Trace GC-TSQ MS (Thermo Scientific, Austin, TX, USA). The system was equipped with a TG–5MS direct capillary. The analysis was conducted under an electron collision system at 70 eV, with 57.5 kPa ionization potential. The injection method utilized was the split mode, operating at a linear flow rate of 36.5 cm/s, and a filament emission current of 60 mA. A 1 µL of CSE was introduced into the mobile phase at a calibration flow velocity of 1 mL/min, which contained 99.9% pure helium. Initially, the column temperature program was maintained at 50 °C for two minutes by an isothermal hold. Following this, the temperature was ramped up to 250 °C at a rate of 5 °C per minute for two minutes. Subsequently, the temperature increased to 300 °C at a rate of 30 °C per minute and held steady for two minutes. In this experiment, the temperatures for the MS and the injector transmission line were meticulously regulated at 270 °C and 260 °C, respectively. To determine which specific chemicals were separated during the study, the ionization spectra were compared with reference data and the library from WILEY 09 and the National Institute of Standards and Technology (NIST14) [108].

#### 4.3.3. Spectrophotometric Evaluation of CSE Phenolic Compounds

For the quantification of total phenolic compounds in the CSE using spectrophotometry, the process began by dissolving one milliliter of the CSE in two milliliters of methanol. Subsequently, 500 μL aliquots of this methanol-extract mixture were taken and combined with 2.5 mL of Folin–Ciocalteu reagent and 2.5 mL of 75 g/L sodium carbonate solution, which were diluted tenfold. The combined solution was then vortexed for 10 s then the tubes containing the mixture were set at 25 °C for a duration of two hours to allow the reaction to proceed. Post incubation period, the absorbance was measured at wavelength 765 nm, with a reagent blank serving as the reference point. The CSE total phenolic content was then measured and represented in milligram of gallic acid equivalents (GAE) per gram of CSE [109].

#### 4.3.4. Spectrophotometric Evaluation of CSE Flavonoid Compounds

A modified AlCl3 colorimetric technique was employed to quantify the total amount of CSE flavonoid compounds. In this procedure, 1 mL of the CSE was placed in 2 mL of methanol in a 10 mL glass flask. Subsequently, a 25 mL volumetric flask was used to prepare a solution containing 7% AlCl3, 5% NaOH, and 5% NaNO3, dissolved in water. From the prepared extract solution, 200 microliters were taken and placed into a sealed glass vial. A further 75 μL of 5% NaNO_3_ was added to the vial, the mixture was let to settle for five minutes. After that, each vial received an addition of 1.25 mL of AlCl_3_ and 0.5 mL of NaOH. The mixtures were subsequently subjected to ultrasonication and incubated for 5 min at ambient temperature. Post incubation period, the absorbance of all solutions, encompassing both the working and standard solutions, was meticulously recorded at 510 nm, utilizing a methanol blank as a reference. The CSE flavonoid content was then measured and represented in milligram of quercetin equivalents (Qu) per gram of CSE [110].

#### 4.3.5. Untargeted Metabolic Analysis for CSE

All untargeted UPLC–ESI–QTOF–MS analyses were performed by the authors specifically for this study. The referenced literature pertains only to the analytical workflow and instrumental parameters, not to previously published datasets. The UHPLC/QTOF-MS technique was conducted as previously reported to explore the phytochemical profile of CSE in ESI (+)/ESI (−) modes. A solution of CSE was initially prepared by dissolving in a MeOH-ACN mixture (2:1). After the sample was subjected to vortex mixing and ultrasonication, an additional 1 mL of solvent was added, and the resulting solution was centrifuged at 10,000 rpm for 5 min before it was analyzed. Similarly, the same protocol was used for blank and quality control samples. The analysis was performed as previously reported on the C18-RP column using acetonitrile, methanol, and water as eluents. For both ionization events, the ion-spray voltage and sprayer capillary were set at +4500/+80 V and 4500/80 V with a mass range of 50–1100 *m*/*z*. The data were gathered and analyzed as previously shown using MS-DIAL (v4.9.221218; RIKEN Center for Sustainable Resource Science, Yokohama, Japan), and Peak view software (v2.2; AB SCIEX, Framingham, MA, USA) [111].

### 4.4. Evaluation of Cytotoxic Activity

A growth inhibition test on Wi38, Hep2, and A549 cell lines was conducted to assess the cytotoxicity of the CSE. Following the previously reported method [112], a 96-well tissue culture plate was filled with 100 µL of a cell suspension with cellular conc. 1 × 10^5^ cells/mL per well. The plate was kept at 37 °C for one day to facilitate cellular attachment. Following the incubation period, the growth material was carefully removed by suction and then washed twice with wash media to remove non-adherent cells. Subsequently, the sample was diluted in RPMI media using a two-fold sequential dilution, and then 100 µL of each concentration was introduced into separate wells. For control, three wells were retained and filled with media without samples and incubated at 37 °C. To assess cell viability20 µL of MTT solution, prepared at a concentration of 5 mg/mL in PBS, was added to each well. To ensure thorough mixing of the MTT and medium, the plate was shaken at 150 rpm for 5 min. Subsequently, the plate was incubated at 37 °C for 240 min to complete the MTT reduction process. After incubation, the suspension was removed, and crystalline formazan was solubilized in 200 µL of DMSO. The plate was then positioned on a shaker set for 5 min to ensure thorough mixing of the formazan with the solvent. The optical density was measured at 560 nm using a SunRise microplate reader (Tecan Group Ltd., Männedorf, Switzerland). Background absorbance at 620 nm was subtracted to obtain a direct measurement of cell density. The outcomes are expressed as a relative percentage compared control value and were determined by identifying the concentration at which cell proliferation was reduced by 50%, denoted as IC_50_, using the software GraphPrism10. The CSE was subjected to triplicate testing.

### 4.5. Antioxidant Profile Assessment

#### 4.5.1. ABTS Radical Scavenging Method

Following the methodology outlined [109], we assessed the scavenging ability of the CSE toward ABTS radical using the ABTS decolorization technique. A mixture with a stable color was prepared by combining potassium persulfate (88 μL, 140 mM) and five mL of 7 mM ABTS, Different amounts of CSE (ranging from 0.5 to 1000 μg/mL) were combined with 100 μL of the ABTS reagent. To measure the antioxidant properties, ascorbic acid was employed as a standard reference, the extent of radical elimination was determined by measuring the absorbance of the reaction product at 734 nm using a spectrophotometer.

#### 4.5.2. H_2_O_2_ Radical Scavenging Method

The hydrogen peroxide reducing power of CSE was assessed using the previous procedure [109]. A solution containing 40 millimolar of hydrogen peroxide was produced in a phosphate buffer with a concentration of 50 mM at pH of 7.4. Different concentrations of the CSE (varying from 0.5 to 1000 μg/mL) were introduced into this solution. Following a 10 min incubation period, absorbance at 230 nm was measured. Butylated hydroxytoluene (BHT) served as a standard reference at the same concentrations. The hydrogen peroxide scavenging activity of the CSE methanolic extract was subsequently assessed using the following calculation formula:

$$\mathrm{Rate}\;\mathrm{of}\;\mathrm{scavenging}\;(H_{2}O_{2})\;=\;\frac{Ai-At}{Ai}\;\times \;100$$

Here, the absorbance of reference (BHT) is denoted by *Ai*, whereas the absorbance of CSE is marked by *At*.

#### 4.5.3. DPPH Radical Scavenging Method

To evaluate the DPPH radical neutralization potential of CSE, the methodology previously described was followed [109]. The DPPH neutralization method operates on the concept that antioxidants can balance the DPPH radical by transferring electrons, resulting in its neutralization and subsequent bleaching of the DPPH solution color. The CSE was tested at concentrations ranging from 0.5 to 1000 μg/mL^−1^ and combined with a 0.66 mM DPPH solution. The solution was placed in an incubator and maintained at a temperature of 25 °C for a duration of 20 min. Following this period, absorbance was assessed at 510 nm with a spectrophotometer, with ascorbic acid serving as the reference standard. The extent of DPPH radical neutralization capacity by the CSE can be assessed by quantifying the reduction in DPPH absorbance. The DPPH percentage reduction in absorbance, indicative of the scavenging activity, is calculated using a calculation formula as follows:

$$\mathrm{DPPH}\;\mathrm{reduction}\;\mathrm{proportion}\;=\;\frac{Abr-Aar}{Abr}\;\times 100$$

where *Abr* represents the optical density recorded before the reaction with the CSE methanolic extract, and *Aar* represents the optical density recorded after the reaction with the CSE methanolic extract.

#### 4.5.4. FRAP Scavenging Method

The FRAP assay was performed based on a standard protocol outlined in [113], with slight adjustments. This technique quantifies CSE capacity to convert ferric ions into ferrous ions. This method relies on the conversion of a ferric iron-TPTZ (2,3,5-triphenyl-1,3,4-triaza-2-azoniacyclopenta-1,4-diene chloride) complex to its ferrous state in an acidic solution. Providing a measure of the CSE’s ability to mitigate oxidation. The FRAP reagent was prepared in 300 mM acetate buffer at pH 3.6 by combining 2.5 mL of a 10 mM TPTZ (2,4,6-tripyridyl-s-triazine) solution in hydrochloric acid with 2.5 mL of a 10 mM hydrated ferric chloride (FeCl_3_) solution. Following activation at ambient temperature, the solution was combined with CSE across a concentration spectrum of 0.5 to 1000 μg mL^−1^. Absorbance at 593 nm was recorded using a spectrophotometer to assess the solution’s FRAP activity. The results were quantified as micromoles of FeSO_4_ equivalents per gram of CSE. For comparison, ascorbic acid served as a control to establish the benchmark for antioxidant activity

#### 4.5.5. Metal Chelating Activity

The metal chelation activity of CSE was determined using the protocol established by Chaves et al., [114]. This method involves the formation of complexes between ferrozine and Fe^2+^ ions. Ferrozine forms a distinct, red-colored complex with Fe^2+^, which allows for the assessment of metal chelation activity. Chelating agents interfere with the binding of ferrous ions to ferrozine, resulting in a diminished red color of the ferrozine-Fe^2+^ complex. In this assay, CSE was combined with a ferrozine solution (0.25 mM, 0.4 mL) and a ferrous sulfate (FeSO_4_) solution (0.1 mM, 0.2 mL) across concentrations from 0.5 to 1000 μg mL^−1^. EDTA served as a reference control. After incubation, the absorbance of the mixture was recorded spectrophotometrically at 562 nm.

#### 4.5.6. CUPRAC Assay

The CUPRAC assay was conducted following the approach outlined by Apak et al. [115] with minor modifications. The CUPRAC reagent was prepared at pH of 7.0 by combining equal quantities of 7.5 mM solution of neocuproine, a 1 M solution of ammonium acetate buffer and 10 mM solution of copper (II) chloride. The CSE was evaluated across concentrations from 0.5 to 1000 μg/mL. For each concentration, 0.2 mL was added to the CUPRAC solution. The reaction mixtures were allowed to incubate at 25 °C for 1 h to ensure complete progression of the reaction. EDTA served as a reference control to evaluate antioxidant capacity. After incubation, the absorbance of the resulting solutions was recorded at 450 nm, and the antioxidant potential of the CSE methanolic extract was quantified and represented as the quantity of EDTA per gram.

### 4.6. Pharmacological Network Analysis

#### 4.6.1. Chemical Structure Retrieval and Target Identification

The most predominant metabolites of CSE identified by GC–MS, HPLC, and UPLC–T-TOF–MS/MS were retrieved from PubChem [116], using compound names, molecular formulas, or SMILES when necessary. Predicted protein targets were obtained using SwissTargetPrediction (http://www.swisstargetprediction.ch, accessed on 3 January 2025) [117,118], which integrates 2D/3D similarity models to assign probability-based targets. To compile pulmonary fibrosis–associated genes, three databases were queried using the terms “pulmonary fibrosis” and “idiopathic pulmonary fibrosis”: GeneCards (https://www.genecards.org, accessed on 9 January 2025), relevance score ≥ 20) [119], DisGeNET (https://www.disgenet.org/, accessed on 13 January 2025) gda score ≥ 0.2) [120], and the Comparative Toxicogenomics Database (CTD) (https://ctdbase.org/, accessed on 15 January 2025) [121]. Genes meeting the inclusion thresholds were integrated, and duplicates removed, to generate a comprehensive PF-related gene list. The predicted CSE targets and PF-associated genes were compared, and overlapping genes were visualized using Venny 2.1 (http://bioinfogp.cnb.csic.es/tools/venny/, accessed on 23 January 2025). These shared genes were considered potential key targets mediating the pharmacological effects of CSE in PF.

#### 4.6.2. PPI Network Construction

To investigate the interactions between intersecting CSE target genes and PF-related genes, a PPI network was constructed based on the STRING 12.5 database [122]. This network comprises both known and predicted protein interactions. The common CSE and PF target genes were then uploaded into STRING with “*Homo sapiens*” as the species. A precision confidence cutoff of 0.700 was used to create the STRING network. All interaction types identified through experimental data, curated databases, and prediction techniques were included in the network, and a PPI network with edges that reflected physical or functional connections was created. The constructed PPI network was retrieved and imported into the Cytoscape 3.8.0 [123], for further analysis. To identify densely connected clusters within the PPI network, the Molecular Complex Detection (MCODE) plugin in Cytoscape 3.8.0 was used. Protein complexes with functional significance may be reflected by MCODE.

#### 4.6.3. GO and Pathway Enrichment Analysis

To gain insights into the interacting genes between CSE and PF, functional enrichment analyses were carried out using g:Profiler [124]. g:Profiler identifies relevant Gene Ontology (GO) terms [125,126], Kyoto Encyclopedia of Genes and Genomes (KEGG) pathways [127], and potential disease correlations by mapping the identified genes to well-annotated genes. The significantly enriched biological processes (BP), cellular components (CC), and molecular functions (MF), along with the KEGG pathways, were visualized using the SRplot [128].

#### 4.6.4. Identification of Target Genes–miRNA and miRNA–lncRNA Interactions

The potential gene-lncRNA connections of *miR-let-7d* were identified using a computational method. This analysis was performed to examine regulatory role of *miR-let-7d* in Mtx-induced PF. For this, the target genes identified by KEGG pathway analysis were first evaluated for *miR-let-7d* interaction using the miRDB database (https://mirdb.org/, accessed on 20 February 2024) [129]. The miRDB database offers insights into microRNA–mRNA regulation networks. Further, DIANA-LncBase v3 database (https://diana.e-ce.uth.gr/lncbasev3, accessed on 20 February 2024) [130], was used to identify lncRNAs that may be targeted by *miR-let-7d*. DIANA-LncBase v3 database is an experimentally validated database of miRNA–lncRNA interactions, and in this study, it was used to investigate the post-transcriptional regulatory axis. Default parameters were used for this analysis, with the species filter limited to *Homo sapiens* and the interaction threshold set at ≥0.07 to guarantee good sensitivity and accuracy.

### 4.7. Evaluation of Antifibrotic Potential in a Pulmonary Fibrosis Animal Model

#### 4.7.1. Animals

The experimental procedures involving animals received approval from the Institutional Animal Ethics Committee of Port Said University Faculty of Science (EBN: PSU.Sci.12, 27 September 2023). All procedures were conducted in accordance with the NIH Guide for the Care and Use of Laboratory Animals (8th edition) and institutional ethical standards. A total of 40 male Wistar rats, weighing between 150 and 200 g, were obtained from the National Research Institute in Cairo, Egypt, and were certified free of known pathogens. All rats were clinically healthy, immunocompetent, wild-type animals with no prior experimental procedures. The animals were housed in standard cages (five rats per cage) with autoclaved wood-shaving bedding at the Port Said University’s animal facility. The animal facility was maintained at 22–25 °C with 50–60% relative humidity under a controlled 12 h light/dark cycle. Rats had free access to standard laboratory chow and filtered tap water ad libitum. All animals underwent a one-week acclimatization period before experimentation to ensure ethical compliance and optimal animal well-being throughout the study.

#### 4.7.2. Experimental Design

The sample size was estimated a priori using GraphPad Prism (version 10) following standard assumptions for comparing two independent means with an unpaired *t*-test (α = 0.05, two-tailed, and 80% power). This yielded a sample size of five animals per subgroup, which reflects a balance between statistical rigor and ethical considerations in accordance with the 3Rs principle. Accordingly, forty rats (n = 40) were randomly assigned, using simple randomization, to four predefined groups, including both disease and treatment arms as well as a vehicle-treated control group, following ARRIVE guidelines. To minimize potential confounders, all animals were housed under identical environmental conditions, and treatments, sample collections, and measurements were performed in a consistent sequence across all groups during the same time frame each day. Cage positions were rotated weekly to avoid location-related effects. Group allocation and treatment administration were performed by personnel who were not involved in the subsequent outcome assessments. In the present study, the Mxt-induced model was selected as an established chemical model of PF because it reliably induces oxidative stress, inflammation, and collagen deposition that mimic key pathological features of PF [36,37,38]. MTX was administered at a dose of 0.5 mg/kg body weight] mg/kg via intraperitoneal injection on day 1 to induce fibrosis. CSE doses were selected based on previously reported safe and pharmacologically effective ranges for plant extracts and preliminary tolerability studies to cover a low, moderate, and high range without causing toxicity. CSE was administered once daily by oral gavage at doses of 50, 100, or 150 mg/kg, starting 24 h after MTX administration. Control groups received equivalent volumes of vehicle.

i Control naïve group (n = 5): Rats received intratracheal injections with saline twice a week (1 mL of normal saline).
ii. PF model group (Mtx model, n = 5): 0.9% NaCl solution was administered with the Mtx (0.5 mg/kg body weight) by intraperitoneal injection twice a week for four weeks according to Mohamed et al. [131].
iii. Control + CSE (n = 5/per dose, total n = 15): Rats were orally treated with CSE at doses of either 50 mg/kg/day dissolved in 0.1 mL of normal saline (CSE 50 mg, n = 5), 100 mg/kg/day (CSE 100 mg, n = 5), or 150 mg/kg/day (CSE 150 mg, n = 5) for four weeks.
iv. CSE-treated PF group (n = 5/per dose, total n = 15): Rats were given Mtx (0.5 mg/kg body weight) intraperitoneally 2 times per week for four weeks, followed by oral administration with CSE at doses of 50 mg/kg/day (CSE 50 mg, n = 5), 100 mg/kg/day (CSE 100 mg, n = 5), or 150 mg/kg/day (CSE 150 mg, n = 5) for four weeks.

All administrations were performed at the same time each morning to avoid circadian variability. Animals were monitored daily for clinical signs, behavior, and body weight. To minimize discomfort, all procedures were performed using gentle handling, and animals showing signs of distress were examined by a veterinarian. Mtx-induced PF has known adverse effects such as reduced activity, ruffled fur, and weight loss; however, no unexpected adverse events or mortality occurred during the study. Humane endpoints were predefined as a loss of >20% body weight, severe respiratory distress, inability to access food or water, or signs of unrelieved pain. No animals reached humane endpoint criteria. Upon completion of the experiment, animals were humanely euthanized under deep anesthesia followed by cervical dislocation, in accordance with institutional and international ethical guidelines. Blood samples were gathered for biochemical examination, while lung tissue was procured for molecular, biochemical, and histopathological assessments from each rat within the various study groups. The primary outcome measure for this study was the degree of pulmonary fibrosis, assessed by quantitative collagen and hydroxyproline content together with histopathological scoring. Secondary outcome measures included inflammatory cytokines (IL-6, HMGB1), oxidative stress biomarkers (MDA, SOD, catalase), extracellular matrix remodeling markers (MMP-1, TGF-β), EMT-associated proteins (α-SMA, E-cadherin), and expression levels of *ncNRFR* and *let-7d*. In all analyses, the individual animal was considered the experimental unit. No a priori inclusion or exclusion criteria were defined for animals or data points. All animals assigned to the study completed the full experimental duration, and no animals or samples were excluded from the analyses. All collected data from all animals (n = 5 per subgroup) were included in the final statistical evaluations. All biochemical measurements, qPCR analyses, and morphometric evaluations were conducted by investigators blinded to the group identity of the samples. Histopathological examinations were performed by an independent pathologist who was unaware of the treatment groups. Data analysis was carried out using coded datasets to maintain blinding until statistical testing was completed.

#### 4.7.3. miRNA Extraction and Processing

A thoraco-abdominal incision was performed to access and extract both lungs from all rats. Subsequently, all specimens underwent double rinsing with cold saline. The left lung was immersed in a 4% paraformaldehyde for histological examination. Meanwhile, the right lungs were kept for biochemical analyses. The collected lungs were homogenized at 4 °C in 0.9% saline and centrifuged for 15 min (Nanosep^®^ 10 kDa; VWR, Fontenay-sous-Bois, France). We identified regulatory miRNAs associated with numerous target genes implicated in lung fibrosis via the InCeDB database (http://gyanxet-beta.com/lncedb/, accessed on 24 February 2024). *Let-7d*, a miRNA linked to cell proliferation and lung fibrosis, was specifically chosen. Following the instructions of the manufacturer, the mini kit RNeasy (Qiagen, Hilden, Germany) was utilized to extract total RNA from the right lung lobes. The NanoDropTM1000 (Thermo Fisher Scientific, Waltham, MA, USA) was utilized to determine the RNA concentration in the sample. An absorbance ratio at A260/A280 that was within the range of 1.8–2.1 was considered indicative of “pure” RNA. For reverse transcription, MiScript II RT PCR kits (Qiagen catalog no. 218161, Hilden, Germany) were employed following the manufacturer’s protocol. Before PCR analysis, the reverse transcription reactions were subsequently kept at −20 °C. These meticulous steps were taken to ensure adherence to ethical and procedural standards, guaranteeing the reliability of the experimental process and the precision of the data obtained.

#### 4.7.4. Assessment of *ncNRFR* and *let-7d* miRNA Expression Using RT-PCR

The qPCR reaction was conducted utilizing the QuantiNove SYBR Green ^®^ PCR Kit (Qiagen, cat. no. 208052) following the manufacturer’s instructions, with prepared cDNA as the template for ncRNA quantification using specific primers (Table S9). Controls (‘No-RT’ and ‘No-template’) were included in each run, and GAPDH served as an endogenous control for normalization using the 2^−ΔΔCT^ method. For *let-7d* miRNA analysis, cDNA templates were synthesized via reverse transcription using the TransScript Reverse Transcriptase protocols AT-101; Beijing TransGen Biotech Co., Ltd., Beijing, China), followed by PCR amplification with the SYBR Green ^®^ kit (Takara Biotechnology Co., Ltd., Dalian, China, Premix Ex Taq™ II) on real-time PCR instrument. The procedure adhered strictly to the manufacturers’ guidelines [132].

#### 4.7.5. Evaluation of Serum Oxidative Stress and Inflammatory Biomarkers

Four weeks later, the animals underwent anesthesia utilizing sodium pentothal (3%). The retro-orbital blood vessels were selected to extract blood samples, after which the animals were subjected to euthanization via exsanguination. Subsequently, the blood samples were treated to separate the serum, which was preserved by freezing until required (−80 °C). Serum levels of IL-6 (Cat.No: MBS355410), TGF-β (Cat.No: MBS011634), HMGB1 (Cat.No: LS-F4039), MMP1(Cat.No: LS-F5522), E-cadherin (Cat.No: MBS3807624), collagen (Cat.No: MBS764752), hydroxyproline (Cat.No: CSB-E08838r), superoxide mutase (SOD) (Cat.No: EKU09415-96T), and catalase (Cat.No: MBS2880214) were detected by ELISA kits. The procedure adhered strictly to the manufacturers’ guidelines.

#### 4.7.6. Histological Studies

##### Tissue Preparation and Histopathological and Immunohistochemical Investigation

Following overnight fixation of the left lung lobes, the lung tissues were rinsed and embedded in paraffin. Next, 3-µm thick paraffin sections of lung tissues were cut using a microtome and used for further histopathological “hematoxylin and eosin (HE) staining for routine examination, or Masson’s trichrome (MT) staining for analysis of the degree of fibrosis” and immunohistochemical staining with alpha-smooth muscle actin (α-SMA) antibody (a mouse monoclonal anti-alpha-smooth muscle actin “α-SMA” antibody, ab212795, Abcam, Cambridge, UK) to detect collagen and smooth muscle fibers. The immunohistochemistry procedures were performed according to our previous report [133].

##### Morphometric Analysis

For histoplanimetric measurements of the degree of lung injury and fibrosis, HE-, MT-, and α-SMA-stained lung sections stained with HE, MT and α-SMA of rats from different studied groups (n = 5 group) were used. To determine the extent of lung injury, the Ashcroft scale was applied based on our previous report [134]. Briefly, digital images from five randomly selected microscopic fields of lung sections/rats stained with MT were captured at ×100 using the BZ-X710 microscope (Keyence, Osaka, Japan) among the different studied groups. Then, a numerical scale was graded from 0 to 8 [135]. Odd-numbered scoring was reported to scale the degree of lung injury in various fields, but even-numbered scoring was recorded if distinguishing between two constitutive odd scorings is difficult. Thereafter, the average score of all grades among the fields and groups was determined and subsequently compared among the groups under investigation. Furthermore, the thickness of the interalveolar septum, the % of the aniline blue^+^ collagen areas, and the % of α-SMA^+^ areas were measured in digital images captured at ×400 from 5 randomly selected fields/rats from immune-stained lung sections from HE, MT, and α-SMA immune-stained lung sections, respectively. For analysis of the thickness of the interalveolar septum, ImageJ software (ver. 1.32j, https://imagej.net/ij/) was used. Next, the % of aniline blue+ collagen area and the% of α-SMA^+^ area were measured in the corresponding captured digital image using a BX analyzer (Keyence, Osaka, Japan). The measurement averages were statistically compared among the groups studied.

#### 4.7.7. Statistical Analysis

Data were analyzed using GraphPad Prism version 10.0 (GraphPad Software, San Diego, CA, USA). For biochemical, molecular, and histopathological parameters, statistical comparisons among groups were performed using one-way ANOVA followed by Tukey’s multiple-comparison post hoc test. Normality of data distribution was assessed using the Shapiro–Wilk test, and homogeneity of variances was evaluated using Levene’s test. All datasets met the assumptions required for parametric testing; therefore, no data transformations or non-parametric analyses were required. Results are presented as mean ± standard error (SE), and statistical significance was set at *p* < 0.05 (**** *p* ≤ 0.0001, *** *p* ≤ 0.001, ** *p* ≤ 0.01, and * *p* ≤ 0.05).

## 5. Conclusions

PF remains a progressive and life-threatening lung disease with limited therapeutic options. In this study, we provide a comprehensive evaluation of the potential antifibrotic activity of CSE using integrated in vitro, in silico, and in vivo approaches. Phytochemical profiling by GC–MS, HPLC, and UPLC–T-TOF–MS/MS revealed a diverse metabolome enriched in terpenes, flavonoids, phenolics, glucosinolates, and amino acid derivatives, many of which have previously documented antioxidant or anti-inflammatory properties. In vitro, CSE demonstrated notable radical-scavenging and metal-chelating activities and displayed differential cytotoxicity between malignant and non-malignant cells. Network pharmacology analysis identified a broad set of predicted interactions between CSE metabolites and PF-related genes, suggesting *ncNRFR, let-7d*, IL-6, and MMP-1 as candidate regulatory nodes. In vivo, CSE administration in the MTX-induced PF model was associated with improvements in oxidative stress, inflammation, extracellular matrix remodeling, EMT markers, and histopathology. CSE also correlated with modulation of *ncNRFR* and *let-7d* expression; however, these findings represent associative changes rather than demonstrated mechanistic interactions. Collectively, our results indicate that CSE mitigates key pathological features of MTX-induced pulmonary fibrosis through the combined influence of multiple biological processes, although the underlying molecular mechanisms remain to be established. The putative involvement of *ncNRFR* and *let-7d* provides a basis for further hypothesis-driven research, including targeted mechanistic studies to determine the causal relevance of this lncRNA–miRNA relationship. Future work should also focus on isolating the active phytochemical constituents and clarifying their discrete and collective roles in antifibrotic activity.

## Figures and Tables

**Table 1:** The list of metabolites detected in the methanolic extract of CSE by GC-MS analysis.

No	RT (min)	Detected Metabolite	Class	Area (%)	Molecular Formula	Molecular Weight
1	6.25	γ-Pyronene	Monoterpene	1.20	C_10_H_16_	136
2	6.56	* cis * -β-Terpineol	Monoterpene	3.21	C_10_H1_8_O	154
3	6.75	* cis * -4-Thujanol	Monoterpene	13.20	C_10_H_18_O	154
4	8.78	(*S*)-(-)-α-Terpineol	Monoterpene	2.53	C_10_H_18_O	154
5	10.35	γ-Terpineol	Monoterpene	20.82	C_10_H_18_O	154
6	11.70	±-Methoxyphenylacetic acid	Sesquiterpene	4.92	C_9_H_10_O_3_	166
7	14.61	Caryophyllene	Sesquiterpene	3.53	C_15_H_24_	204
8	16.38	γ-Elemene	Diterpene hydrocarbon	2.40	C_15_H_24_	204
9	24.18	Neophytadiene	Fatty acid	1.49	C_20_H_38_	278
10	26.41	n-Hexadecanoic acid	Fatty acid ester	8.50	C_16_H_32_O_2_	256
11	28.65	(*Z*, *Z*)-9,12-Octadecadienoic acid methyl ester	Fatty acid ester	1.30	C_19_H_34_O_2_	294
12	28.82	10-Octadecenoic acid, methyl ester	Neutral lipid	2.10	C_19_H_36_O_2_	296
13	29.56	1-Monolinolenin	Steroid	10.80	C_21_H_36_O_4_	352
14	32.57	Androst-5,7-dien-3-ol-17-one	Alkaloid	1.70	C_19_H_26_O_2_	286
15	35.82	Dasycarpidan-1-methanol, acetate	Alkaloid	1.20	C_20_H_26_N_2_O_2_	326
16	38.79	10-Methoxycoryn-18-en-17-yl acetate	Flavonoid	1.96	C_22_H_28_N_2_O_3_	368
17	39.81	3′,4′,7-Trimethoxyquercetin	Hydrocarbon	1.48	C_18_H_16_O_7_	344
18	40.1	Dotriacontane	Sterol	2.06	C_32_H_66_	450
19	40.15	Stigmast-5-en-3-ol	Flavonoid	1.90	C_29_H_50_O	414
20	40.57	Lucenin II	Neutral lipid	1.96	C_27_H_30_O_16_	610
21	40.84	(*Z*, *Z*)-9-Hexadecenoic acid, 9-octadecenyl ester	Hydrocarbon	1.14	C_34_H_64_O_2_	504
22	41.01	3-Ethyl-5-(2-ethylbutyl)-octadecane	Phenolic	5.51	C_26_H_54_	366
23	41.96	Isochiapin B	Neutral lipid	3.54	C_19_H_26_O_6_	350
24	43.05	Trilinolein	Phenolic	1.55	C_57_H_98_O_6_	878

**Table 2:** The major metabolites detected in the CSE using UPLC/T-TOF in ESI (+) and ESI (−) ionization modes.

Title	RT (min)	Precursor (*m*/*z*)	Area	Error (PPM)	Adduct	Reference (*m*/*z*)	Formula	Classification
N, N-Dimethylglycine	1.00	104.1072	7,232,068	−2.9	[M+H]^+^	104.0706	C_4_H_9_NO_2_	Amino acid derivative
Sinapoyl malate	1.02	339.0699	1,667,280	6.1	[M−H]^−^	339.0721	C_15_H_16_O_9_	Phenolic acid
3-(4-hydroxy-3,5-dimethoxyphenyl)propenoic acid	1.02	223.0624	815,302	−5	[M−H]^−^	223.0612	C_11_H_12_O_5_	Phenolic acid
D-(+)-Raffinose	1.06	503.1620	3,052,157	0.2	[M−H]^−^	503.1618	C_18_H_32_O_16_	Carbohydrate
Maltotriose	1.09	505.1754	1,814,235	0	[M+H]^+^	505.1763	C_18_H_32_O_16_	Carbohydrate
Sucrose	1.11	341.1086	8,841,210	0.9	[M−H]^−^	341.1089	C_12_H_22_O_11_	Carbohydrate
Melibiose	1.12	343.1241	3,607,872	−0.6	[M+H]^+^	343.1235	C_12_H_22_O_11_	Carbohydrate
Xanthosine-5′-monophosphate	1.12	365.1061	801,786	0.5	[M+H]^+^	365.0493	C_10_H_13_N_4_O_9_P	Nucleotide
Glycine-Betaine	1.13	118.0855	328,778	0.8	[M+H]^+^	118.0863	C_5_H_11_NO_2_	Amino acid derivative
Kaempferol-3-Glucuronide	1.21	461.1288	429,942	3.9	[M−H]^−^	461.0725	C_21_H_18_O_12_	Flavonoid glycoside
Scoulerin	1.33	328.1689	216,477	16.5	[M+H]^+^	328.1543	C_19_H_21_NO_4_	Alkaloid
Benzyl glucosinolate	1.48	408.0423	38,789,412	0.3	[M−H]^−^	408.0428	C_14_H_19_NO_9_S_2_	Glucosinolate
L-Tryptophan	2.75	205.0975	1,248,671	−0.6	[M+H]^+^	205.0971	C_11_H_12_N_2_O_2_	Amino acid
2′-Deoxyuridine-5′-triphosphate	5.17	467.1586	381,791	0.6	[M−H]^−^	466.9663	C_9_H_15_N_2_O_14_P_3_	Nucleotide
Trigonelline	5.75	138.0548	232,065	0.4	[M+H]^+^	138.0549	C_7_H_7_NO_2_	Alkaloid
3-Indoxyl sulfate	5.82	212.0004	388,017	7.3	[M−H]^−^	212.0023	C_8_H_7_NO_4_S	Indole derivative
Acacetin-7-O-neohesperidoside	10.14	593.3146	201,047	−0.1	[M+H]^+^	593.1865	C_28_H_32_O_14_	Flavonoid glycoside
3′-methoxy-4′,5,7-trihydroxyflavonol	19.68	317.1152	750,110	−0.4	[M+H]^+^	317.0656	C_16_H_12_O_7_	Flavonol
2′-Deoxycytidine	19.94	228.2310	866,922	5.9	[M+H]^+^	228.0979	C_9_H_13_N_3_O_4_	Nucleotide
3 5 7-trihydroxy-4′-methoxyflavone	21.51	301.1419	630,989	0.7	[M+H]^+^	301.0707	C_16_H_12_O_6_	Flavonol
rosmarinic acid	22.13	359.1499	691,351	3.6	[M−H]^−^	359.0772	C_18_H_16_O_8_	Phenolic acid

## Data Availability

The original contributions presented in this study are included in the article/Supplementary Material. Further inquiries can be directed to the corresponding author.

## Supplementary Materials

The following supporting information can be downloaded at: https://www.mdpi.com/article/10.3390/ph18121820/s1, Figure S1. GC–MS chromatogram of the methanolic extract of CSE. Figure S2–25. Mass fragmentation pattern of γ-Pyronene (RT = 6.25 min), cis-β-Terpineol (RT = 6.56 min), cis-4-Thujanol (RT = 6.75 min), (S)-(-)-α-Terpineol (RT = 8.78 min), γ-Terpineol (RT = 10.35 min), ±-Methoxyphenylacetic acid (RT = 11.70 min), Caryophyllene (RT = 14.61 min), γ-Elemene (RT = 16.38 min), Neophytadiene (RT = 24.18 min), n-Hexadecanoic acid (RT = 26.41 min), (Z,Z)-9,12-Octadecadienoic acid methyl ester (RT = 28.65 min), 10-Octadecenoic acid methyl ester (RT = 28.82 min), 1-Monolinolenin (RT = 29.56 min), Androst-5,7-dien-3-ol-17-one (RT = 32.57 min), Dasycarpidan-1-methanol acetate (RT = 35.82 min), 10-Methoxycoryn-18-en-17-yl acetate (RT = 38.79 min), 3′,4′,7-Trimethoxyquercetin (RT = 39.81 min), Dotriacontane (RT = 40.10 min), Stigmast-5-en-3-ol (RT = 40.15 min), Lucenin II (RT = 40.57 min), (Z,Z)-9-Hexadecenoic acid, 9-octadecenyl ester (RT = 40.84 min), 3-Ethyl-5-(2-ethylbutyl)-octadecane (RT = 41.01 min), Isochiapin B (RT = 41.96 min), Trilinolein (RT = 43.05 min), respectively. Figure S26. HPLC analysis showing detected flavonoids, phenolics, fat-soluble vitamins, and water-soluble vitamins. Figure S27. UPLC/T-TOF analysis: TIC in ESI+ and ESI– modes; %TPA of metabolite classes. Figure S28. Integrated overview of CSE metabolome across GC-MS, HPLC, and UPLC-ESI-QTOF platforms. Figure S29. Antioxidant potential of positive controls (ABTS, DPPH, H_2_O_2_, FRAP, CUPRAC, metal chelation). Figure S30. Complete network of CSE target genes. Figure S31. IL6 as a binding target for miRNA let-7d (3′UTR sequence). Figure S32. MMP1 as a binding target for miRNA let-7d (3′UTR sequence). Table S1. Metabolites detected in methanolic extract of CSE by HPLC analysis. Table S2. Metabolites detected in CSE by UPLC/T-TOF–MS/MS (positive ionization mode). Table S3. Metabolites detected in *C. endivia* extract by UPLC/T-TOF–MS/MS (negative ionization mode). Table S4. Raw CSE-predicted target genes (n = 4307) generated by SwissTargetPrediction, including duplicates prior to filtering. Table S5. Raw PF-associated gene entries (n = 276,411) aggregated from GeneCards, DisGeNET, and CTD, including duplicates prior to filtering. Table S6. Complete STRING-derived PPI network for the shared CSE–PF targets (927 nodes and 12,588 edges), including all interaction scores and evidence channels prior to clustering. Table S7. Complete g:Profiler enrichment results for the shared CSE–PF targets, including all GO terms (BP, CC, MF) and KEGG pathways identified prior to selection of top-ranked categories. Table S8. lncRNAs predicted to interact with hsa-let-7d-5p (DIANA-lncBase v3.0). Table S9. List of gene primers used for RT-qPCR.
