# Supplementary material for: Integrative Metabolomic, Network Pharmacology, and Experimental Evidence for Lepidium sativum Seed Extract as a Natural Modulator of Pulmonary Fibrosis via the ncNRFR/Let-7d Regulatory Pathway
Source: Pharmaceuticals (Basel). 2025 Nov 28;18(12):1820. doi: 10.3390/ph18121820 (PMC13134761; doi:10.3390/ph18121820)
Supplement: Supplementary file 1 [file pharmaceuticals-18-01820-s001.zip › Supporting Information (10)-ES-revised.pdf]

Supporting Information

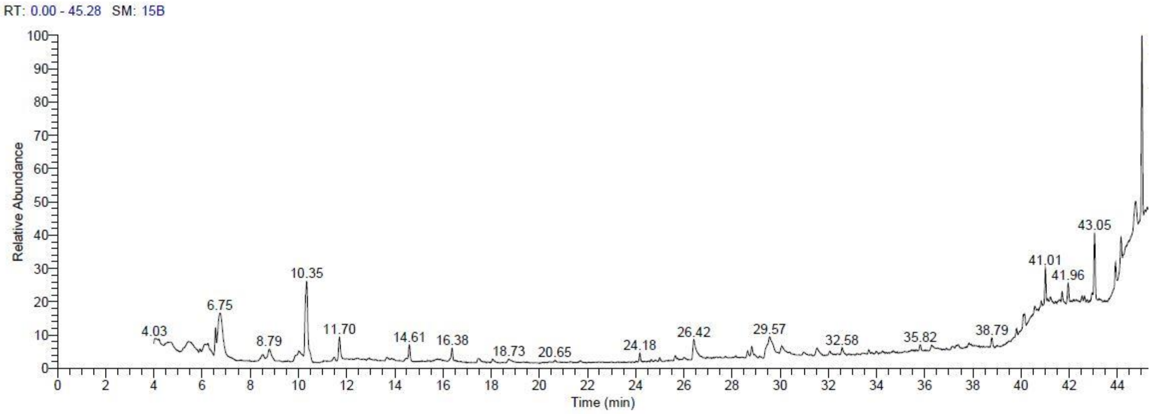

Figure S1: GC-MS chromatogram of the methanolic extract of CSE.

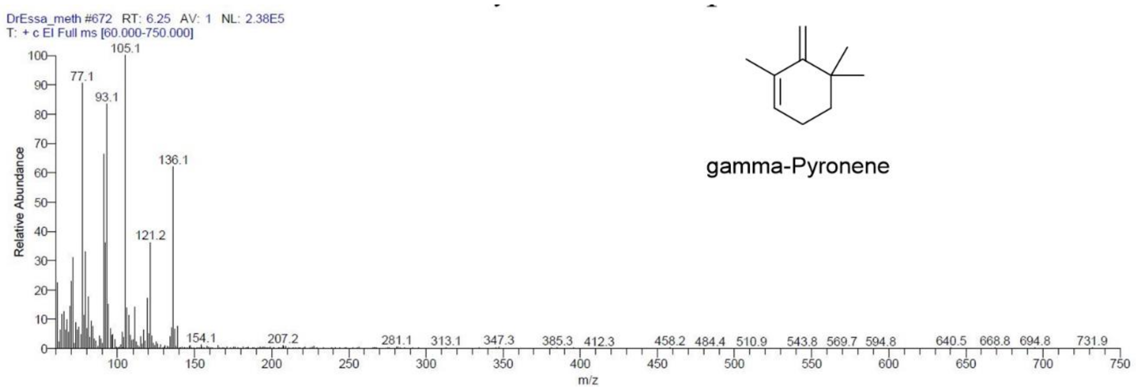

Figure S2: Mass fragmentation pattern of γ-Pyronene with retention time (RT=6.25 min).

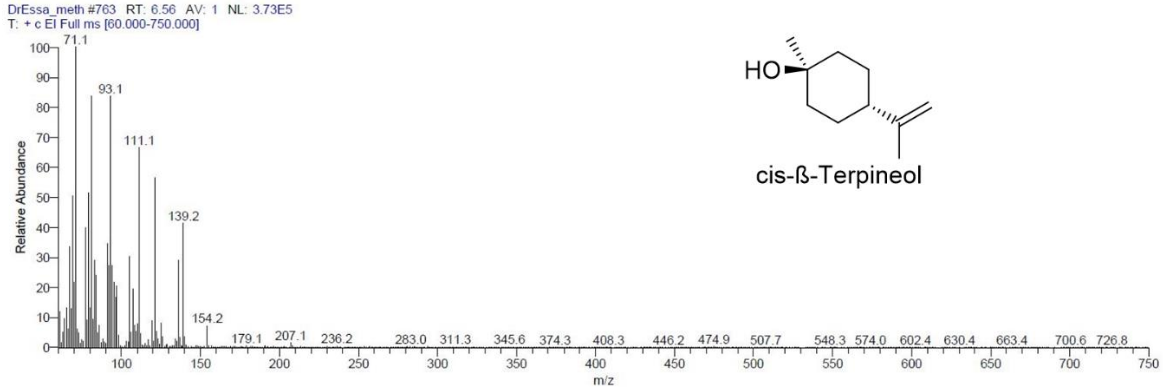

Figure S3: Mass fragmentation pattern of with cis-β-Terpineol retention time (RT=6.56 min).

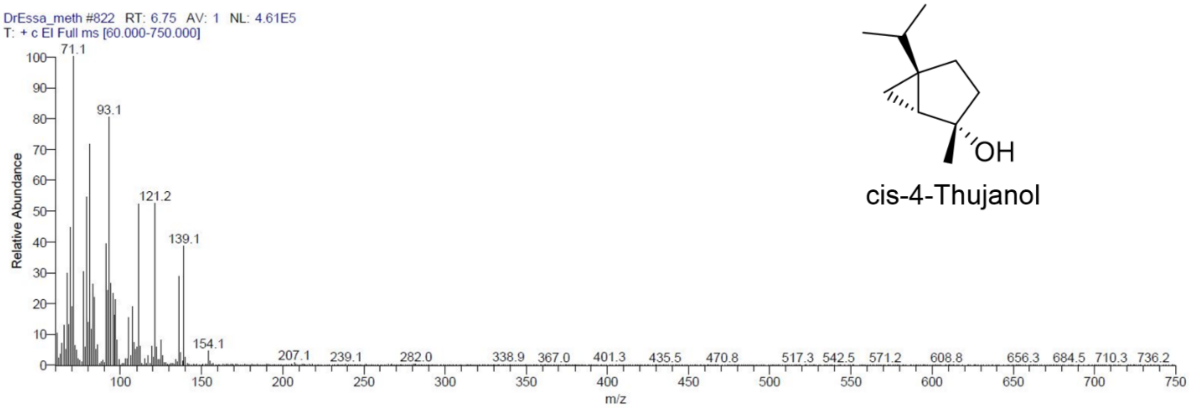

Figure S4: Mass fragmentation pattern of cis-4-Thujanol with retention time (RT=6.75 min).

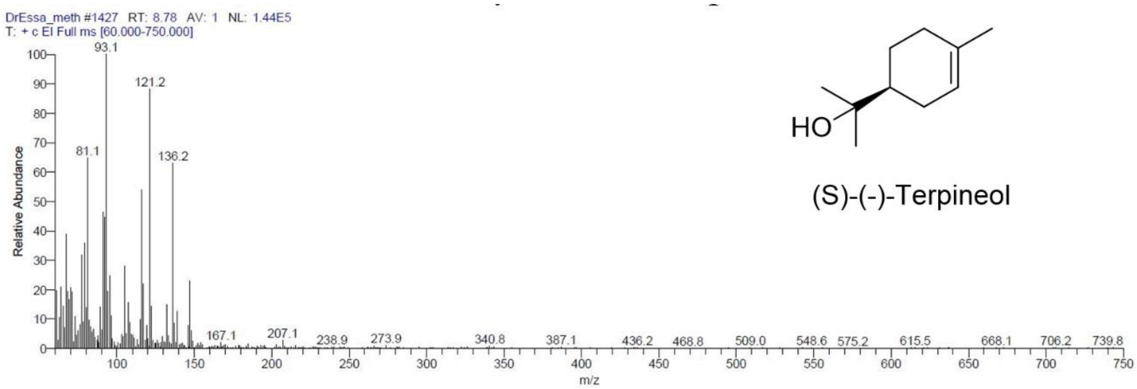

Figure S5: Mass fragmentation pattern of (S)-(-)-α-Terpineol with retention time (RT=8.78 min).

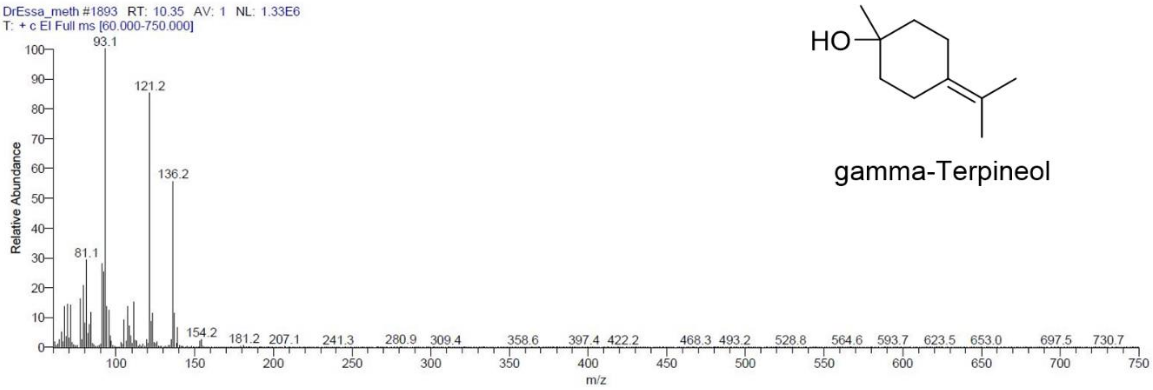

Figure S6: Mass fragmentation pattern of γ-Terpineol with retention time (RT=10.35 min).

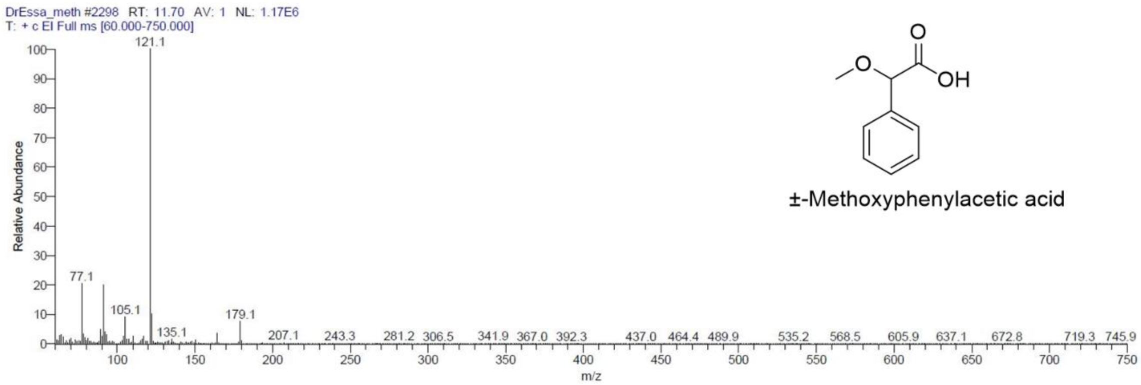

Figure S7: Mass fragmentation pattern of ±-Methoxyphenylacetic acid with retention time (RT=11.70 min).

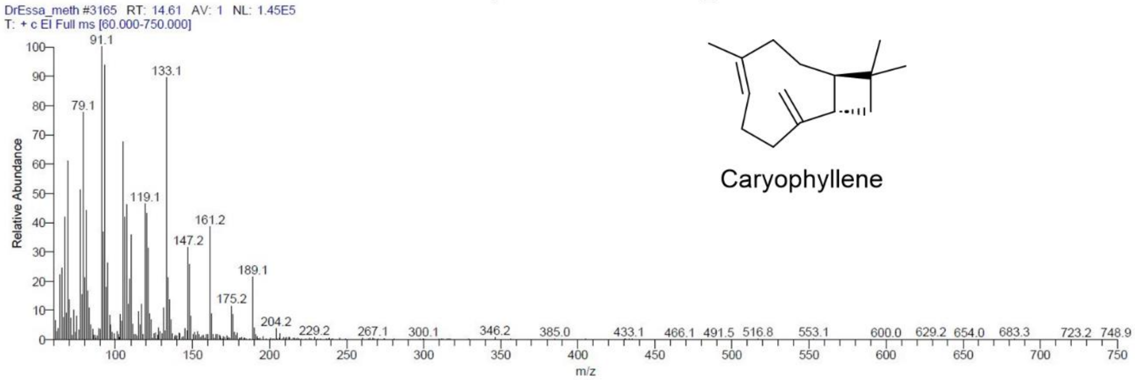

Figure S8: Mass fragmentation pattern of Caryophyllene with retention time (RT=14.61 min).

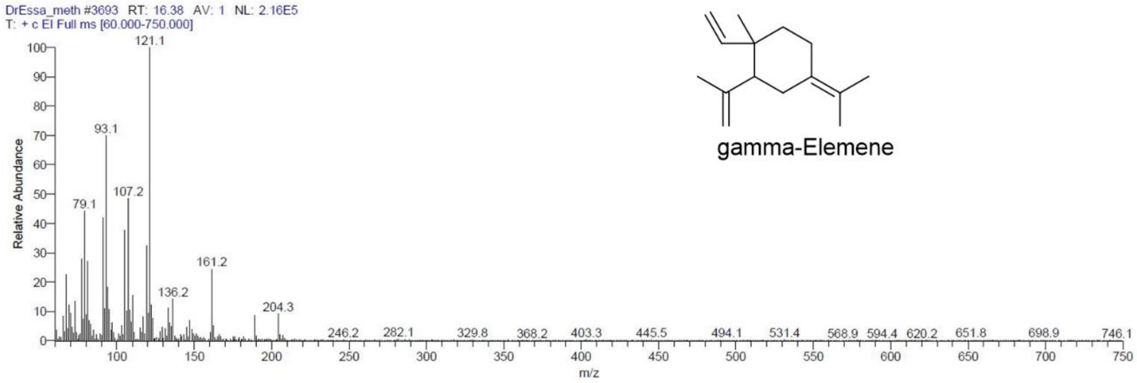

Figure S9: Mass fragmentation pattern of γ-Elemene with retention time (RT=16.38 min).

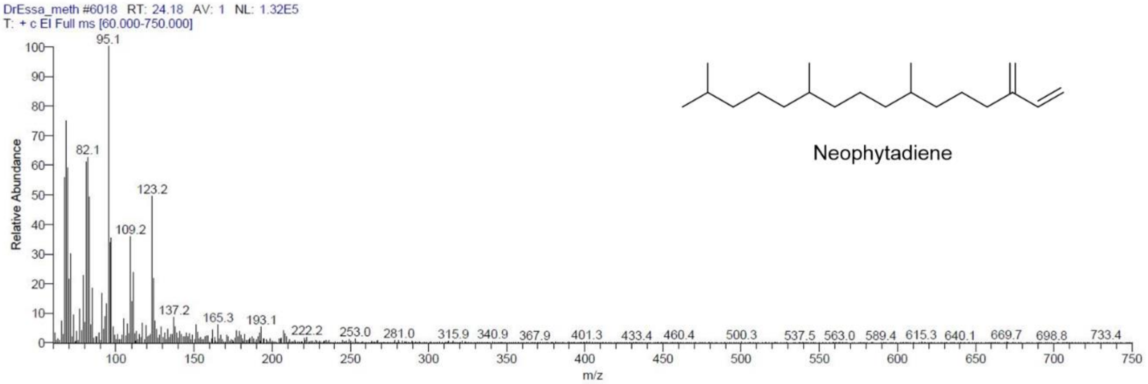

Figure S10: Mass fragmentation pattern of Neophytadiene with retention time (RT=24.18 min).

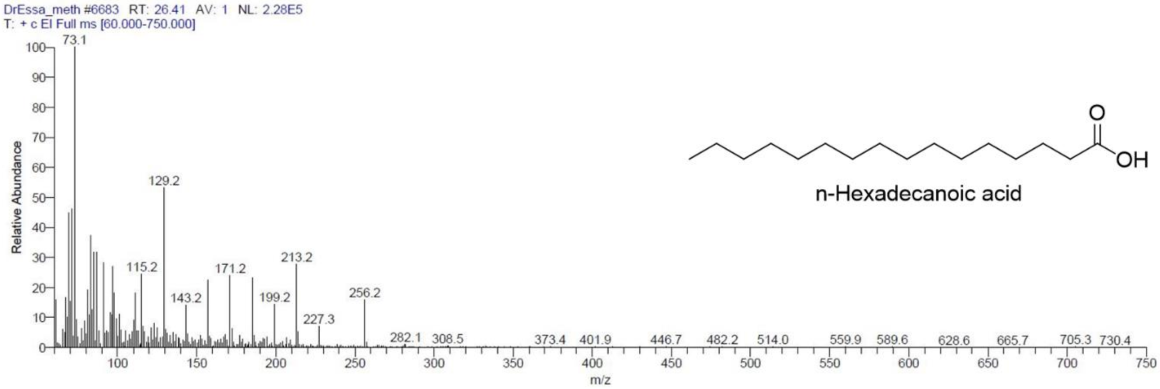

Figure S11: Mass fragmentation pattern of n-Hexadecanoic acid with retention time (RT=26.41 min).

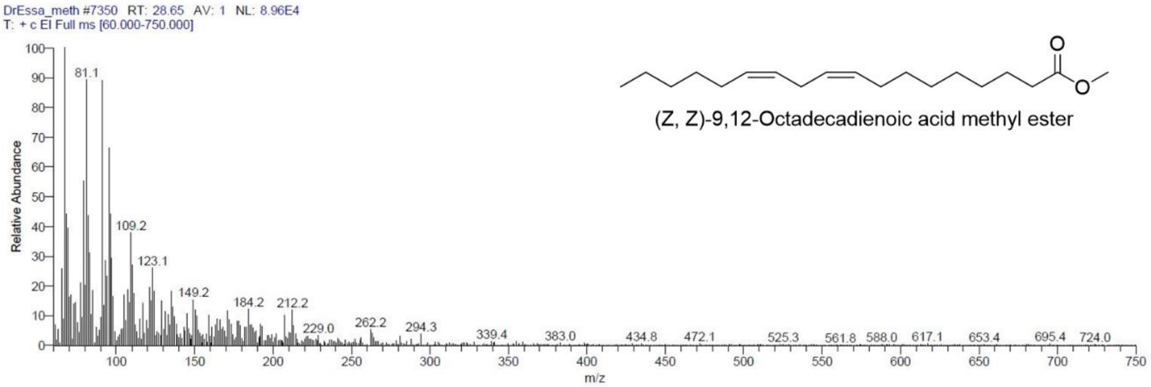

Figure S12: Mass fragmentation pattern of (*Z*, *Z*)-9,12-Octadecadienoic acid methyl ester with retention time (RT=28.65 min).

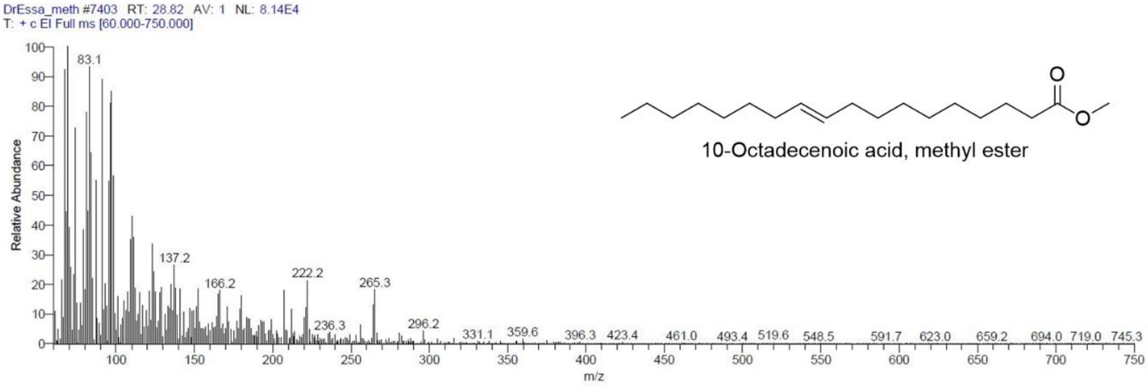

Figure S13: Mass fragmentation pattern of 10-Octadecenoic acid, methyl ester with retention time (RT=28.82 min).

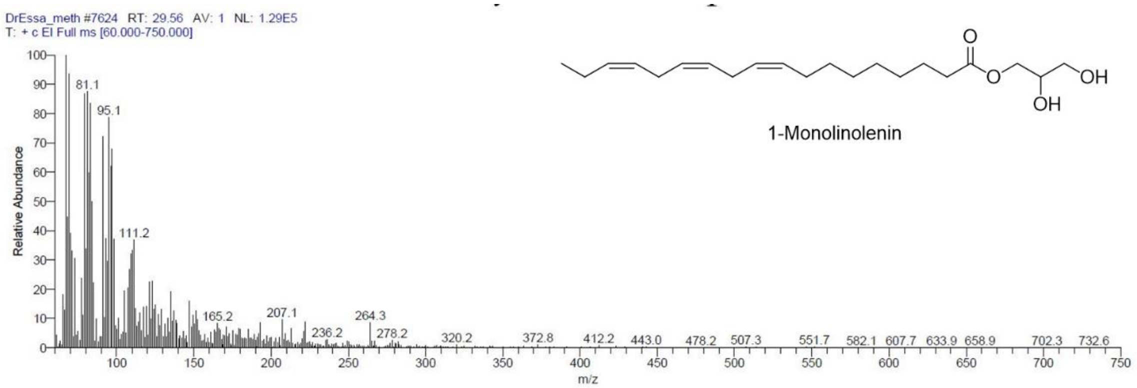

Figure S14: Mass fragmentation pattern of 1-Monolinolenin with retention time (RT=29.56 min).

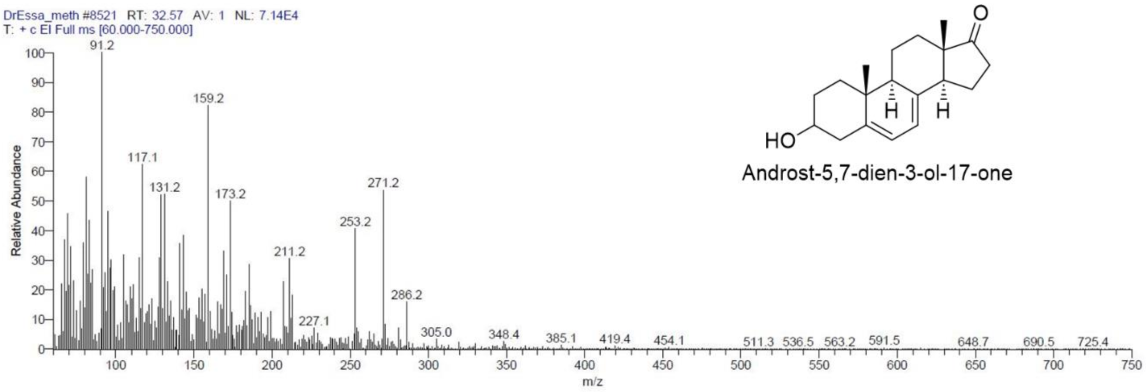

Figure S15: Mass fragmentation pattern of Androst-5,7-dien-3-ol-17-one with retention time (RT=32.57 min).

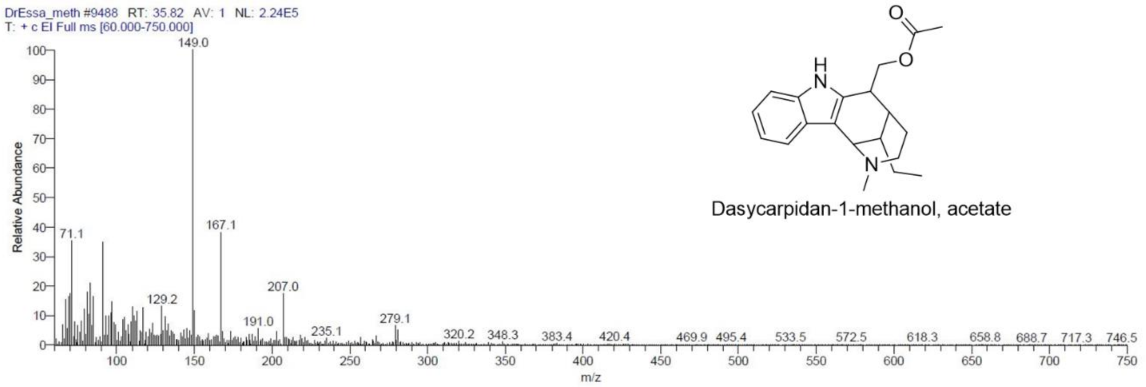

Figure S16: Mass fragmentation pattern of Dasycarpidan-1-methanol, acetate with retention time (RT=35.82 min).

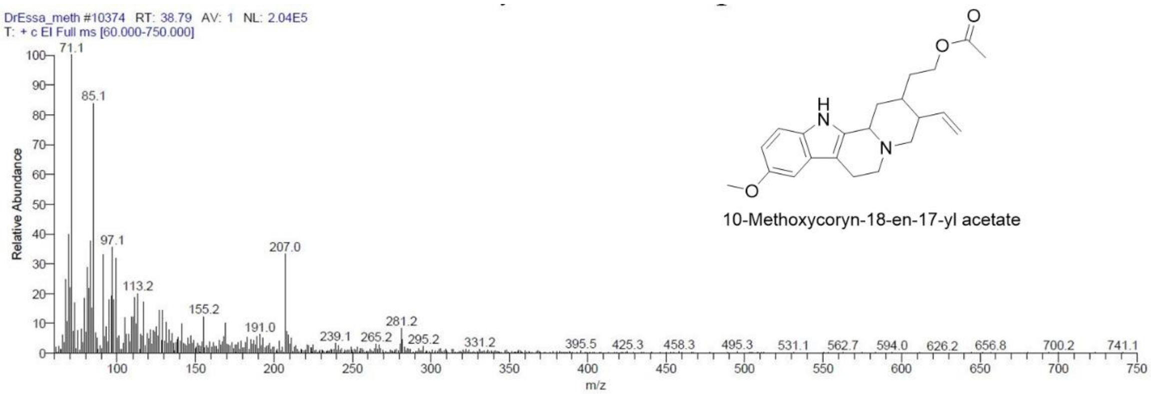

Figure S17: Mass fragmentation pattern of 10-Methoxycoryn-18-en-17-yl acetate with retention time (RT=38.79 min).

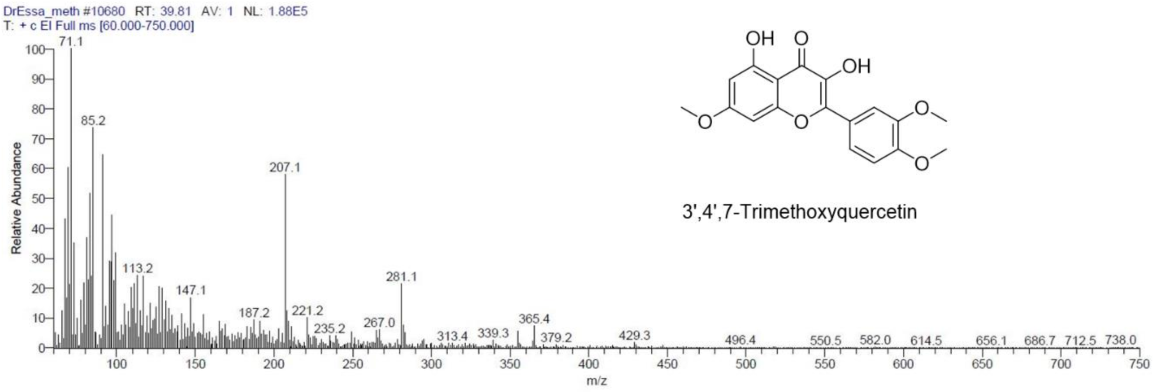

Figure S18: Mass fragmentation pattern of 3',4',7-Trimethoxyquercetin with retention time (RT=39.81 min).

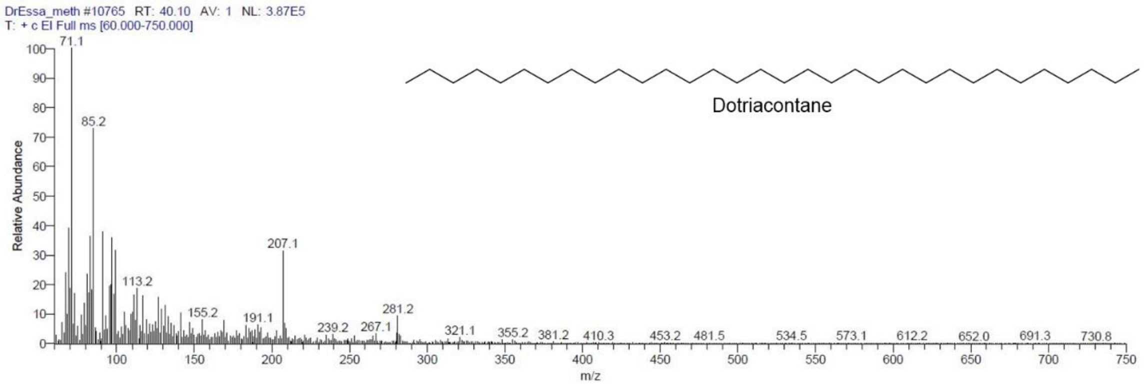

Figure S19: Mass fragmentation pattern of Dotriacontane with retention time (RT=40.10 min).

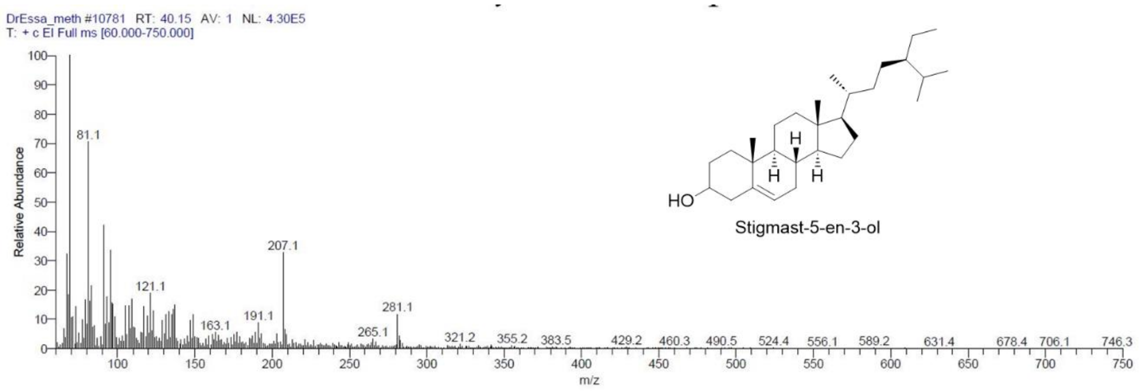

Figure S20: Mass fragmentation pattern of Stigmast-5-en-3-ol with retention time (RT=40.15 min).

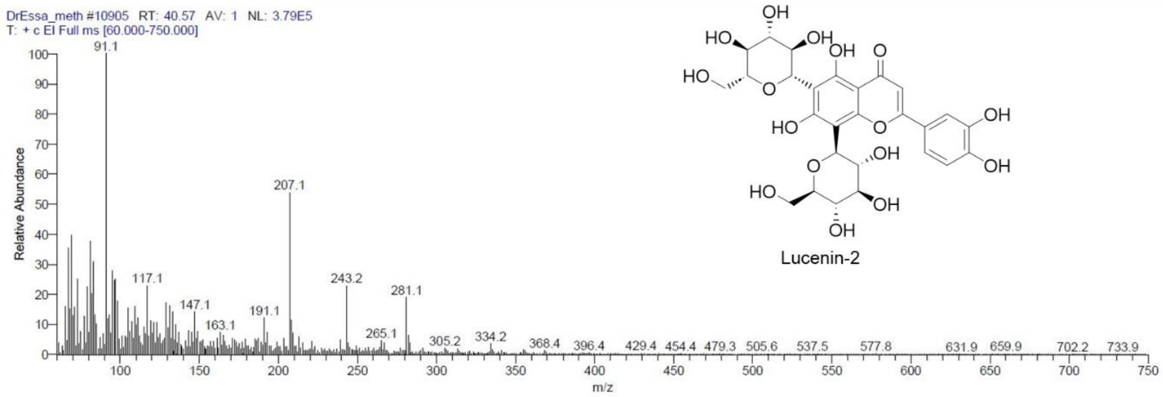

Figure S21: Mass fragmentation pattern of Lucenin II with retention time (RT=40.57 min).

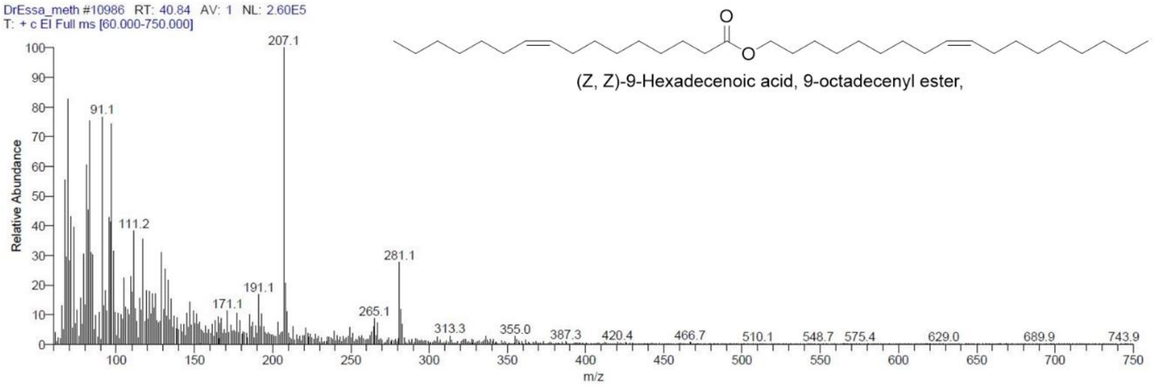

Figure S22: Mass fragmentation pattern of (*Z*, *Z*)-9-Hexadecenoic acid, 9-octadecenyl ester with retention time (RT=40.84 min).

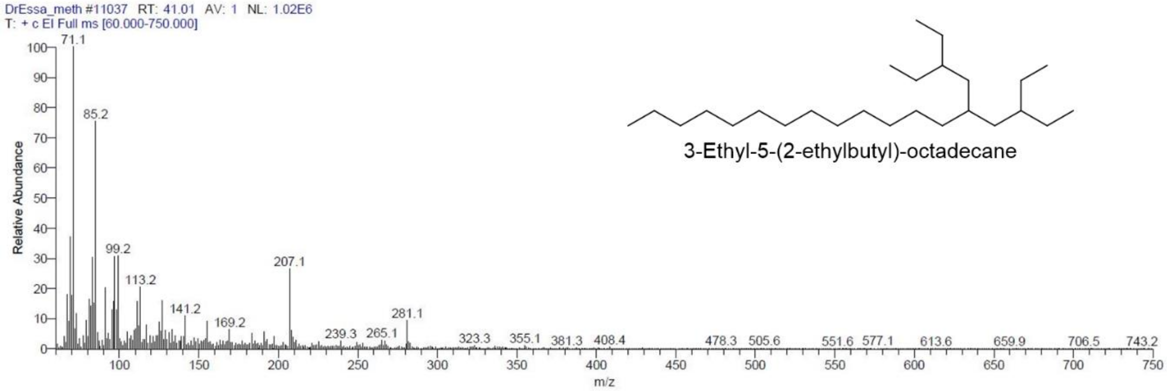

Figure S23: Mass fragmentation pattern of 3-Ethyl-5-(2-ethylbutyl)-octadecane with retention time (RT=41.01 min).

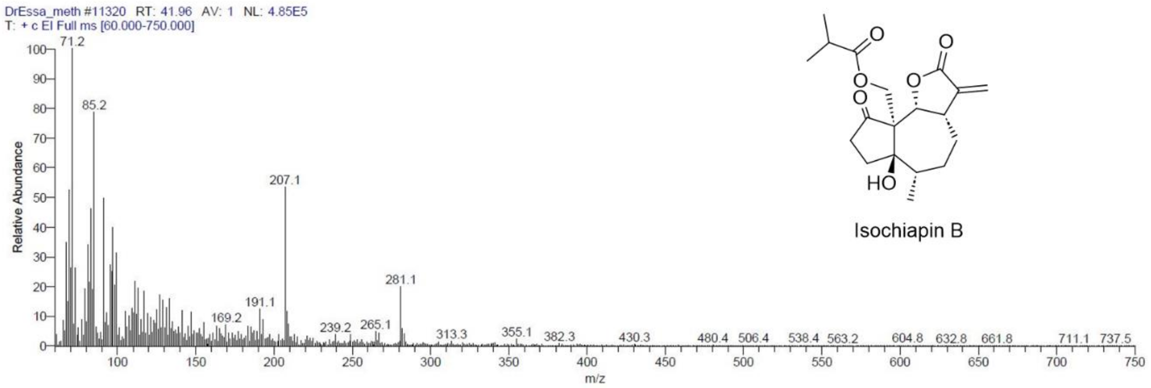

Figure S24: Mass fragmentation pattern of Isochiapin B with retention time (RT=41.96 min).

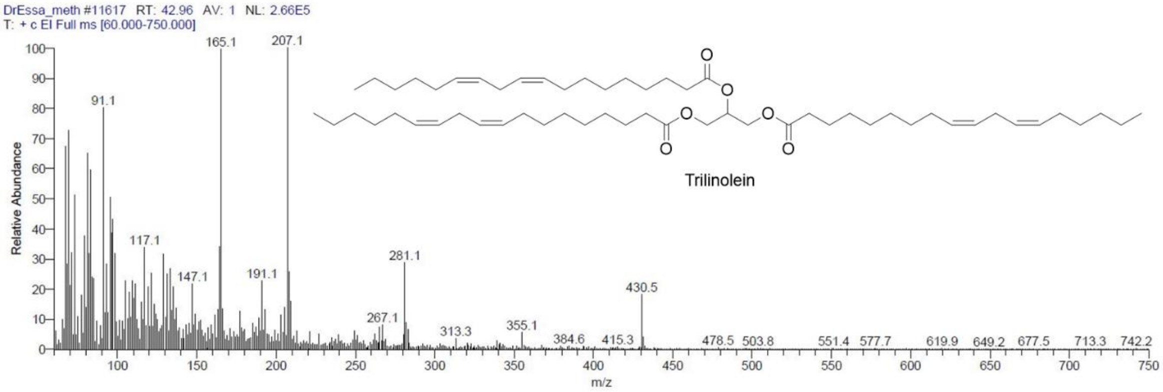

Figure S25: Mass fragmentation pattern of Trilinolein with retention time (RT=43.05 min).

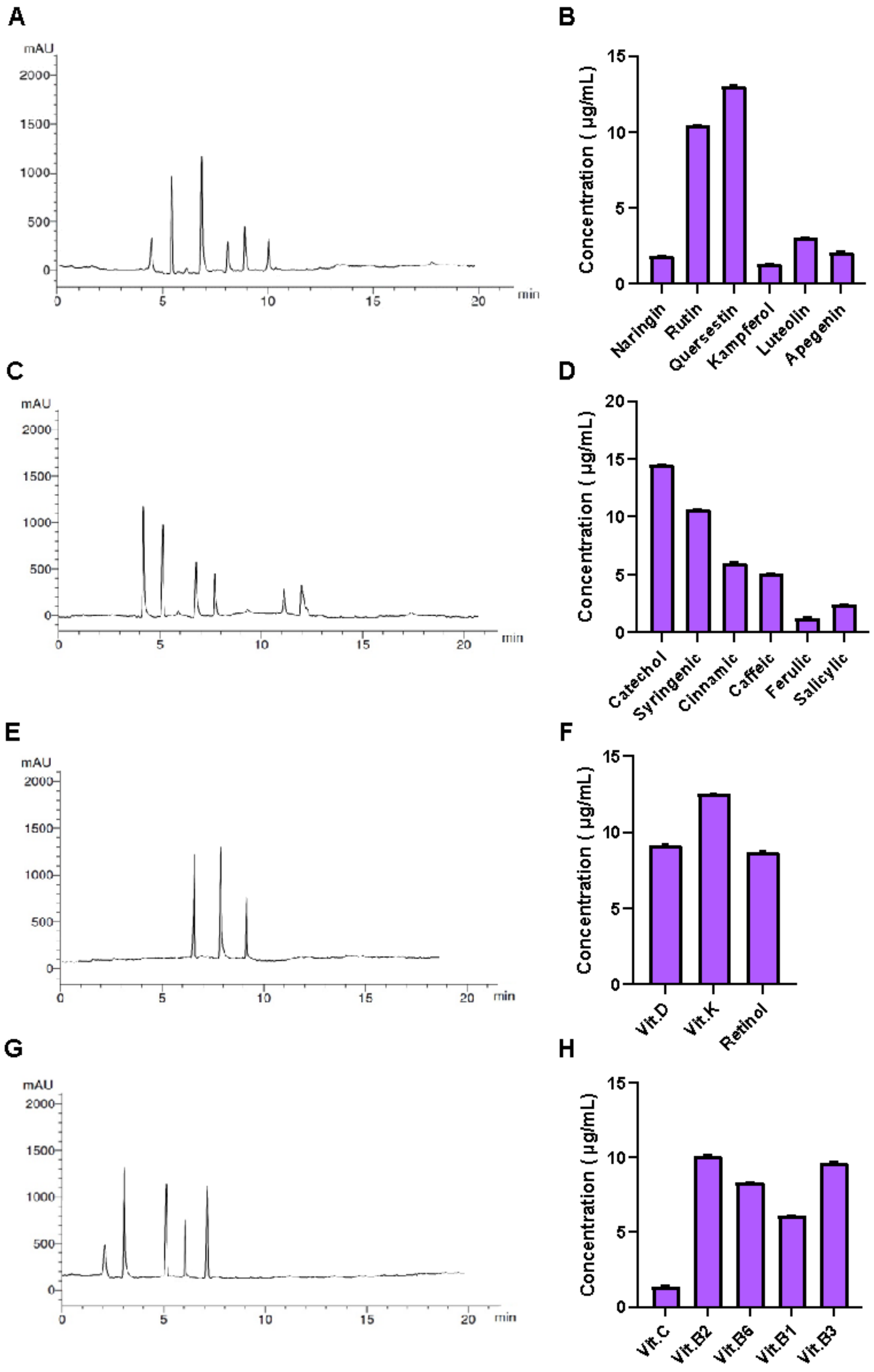

Figure S26: HPLC analysis of CSE extract showing the detected flavonoids (**A, B**), phenolics (**C, D**), fat-soluble vitamins (**E, F**), and water-soluble vitamins (**G, H**) metabolites in CSE.

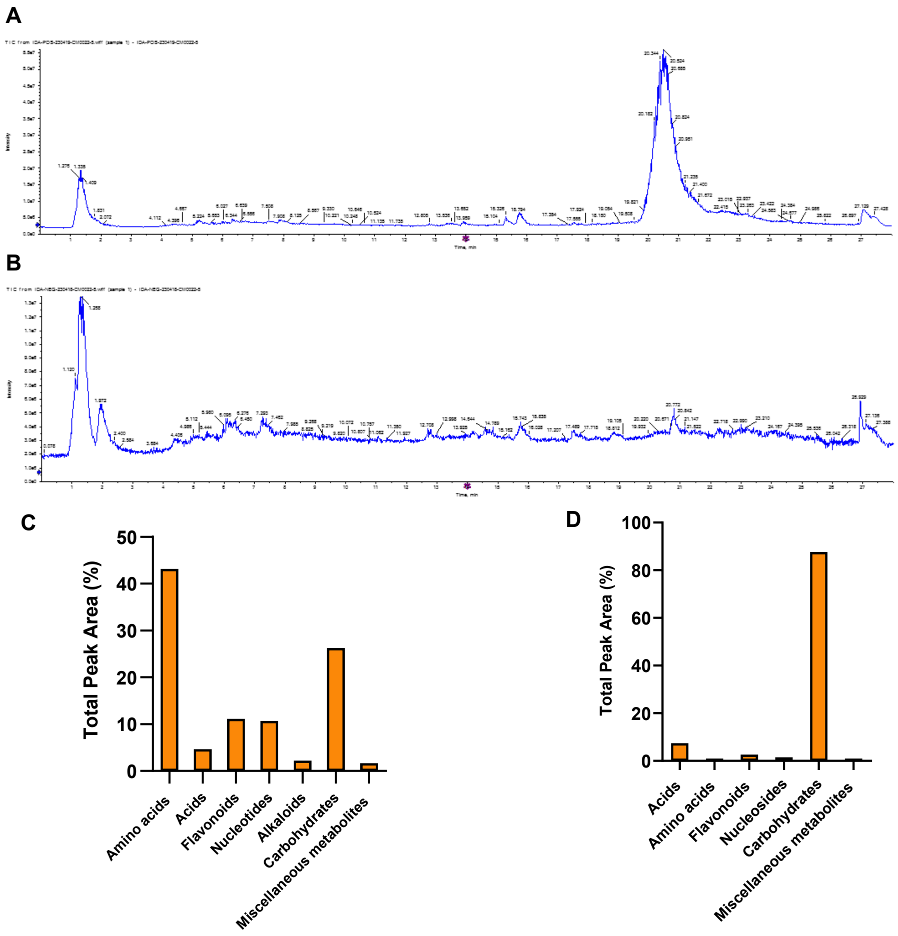

Figure 27: The UPLC/T-TOF assessment of the CSE showing the total ion chromatogram at ESI (+) **(A)** and ESI (-) **(B)** ionization modes. The various classes of metabolites detected (% TPA) in the ESI (+) **(C)** and ESI (-) ionization modes **(D)**.

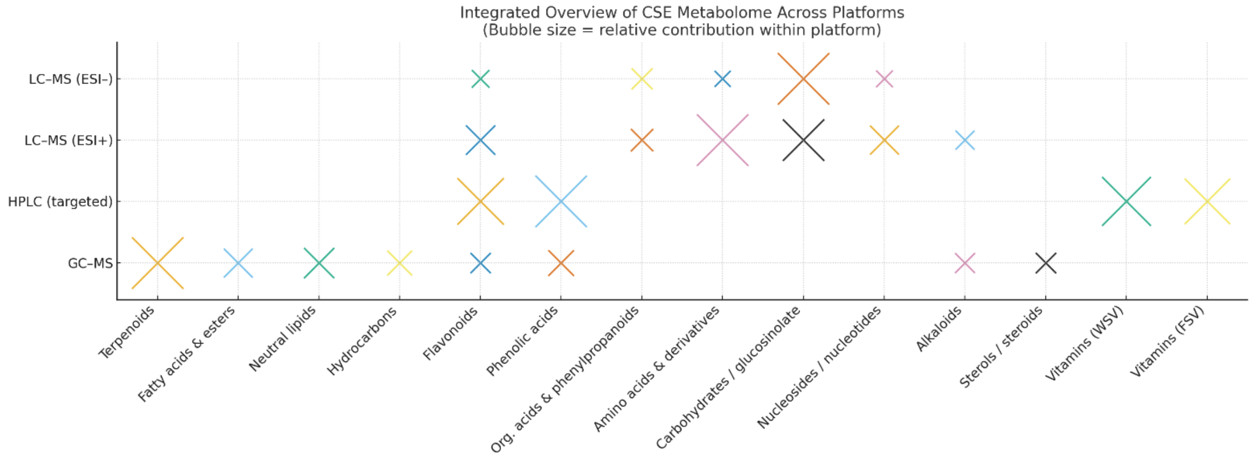

Figure S28. Integrated overview of the *CSE* metabolome across analytical platforms. Bubble size reflects the relative contribution of each metabolite class within a platform (normalized to the largest class per row). GC–MS highlights volatile lipophilic classes (terpenoids, fatty acids, neutral lipids, hydrocarbons), HPLC quantifies polar bioactives (phenolic acids, flavonoids, water- and fat-soluble vitamins), and UPLC–ESI–QTOF maps ionizable metabolites (ESI+, amino-acid derivatives, nucleosides; ESI–, phenylpropanoid conjugates, oligosaccharides, glucosinolates). Overlap in classes (e.g., flavonoids/phenylpropanoids) indicates cross-platform consistency, while mode-specific enrichments illustrate complementary coverage.

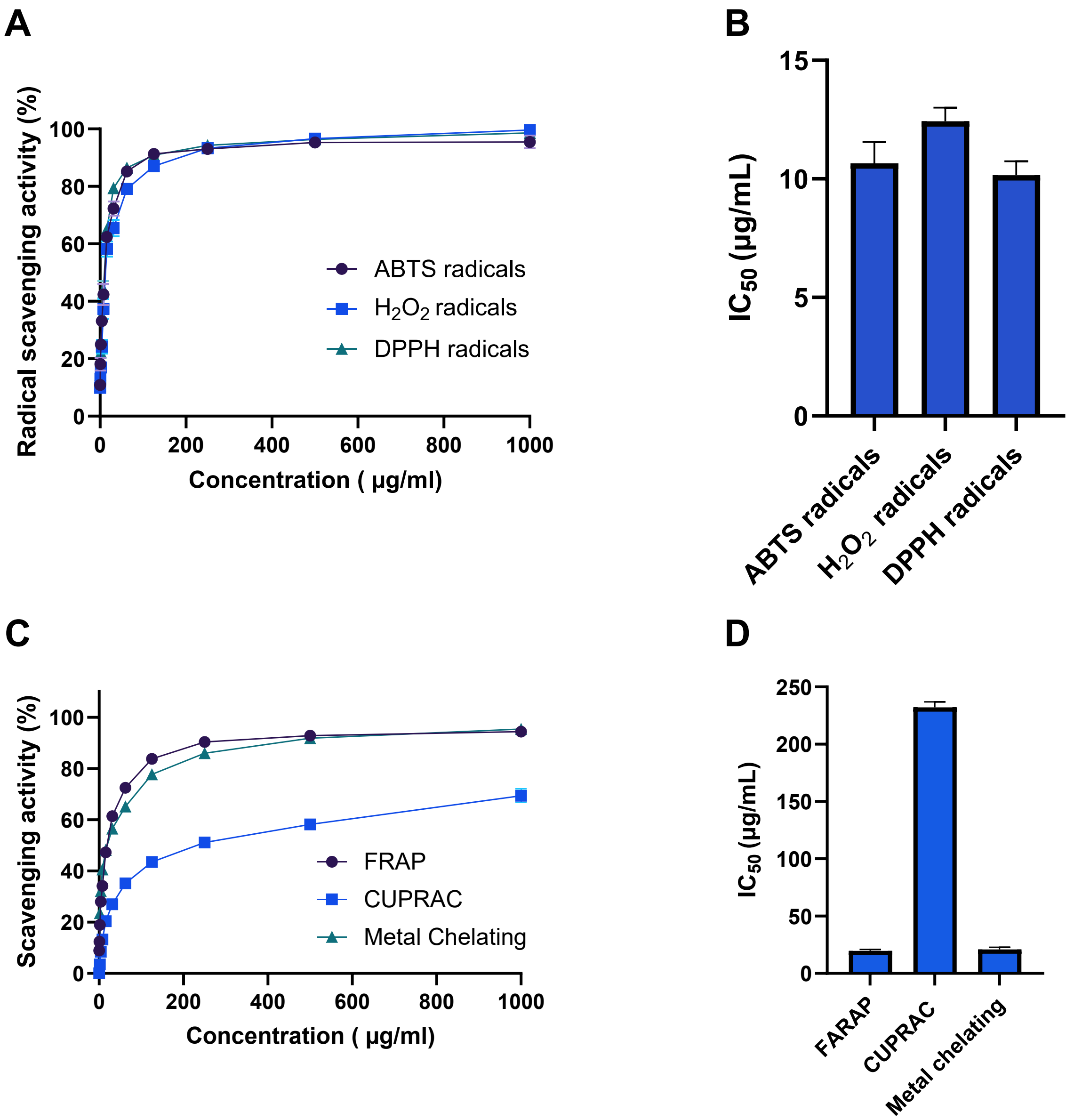

Figure S29: The *in vitro* antioxidant potential of the positive controls (ascorbic acid, BHT, EDTA) as examined by scavenging activity (%) toward ABTS, H_2O_2, and DPPH radicals (**A, B**), and copper and iron ions, and metal chelating (**C, D**). The presented data was expressed as mean ± S.D in triplicate. Ascorbic acid was utilized as a positive control for ABTS, DPPH, and FRAP assays. EDTA was utilized as a positive control for CUPRAC and metal chelating assay. BHT was utilized as a positive control for H_2O_2 assay.

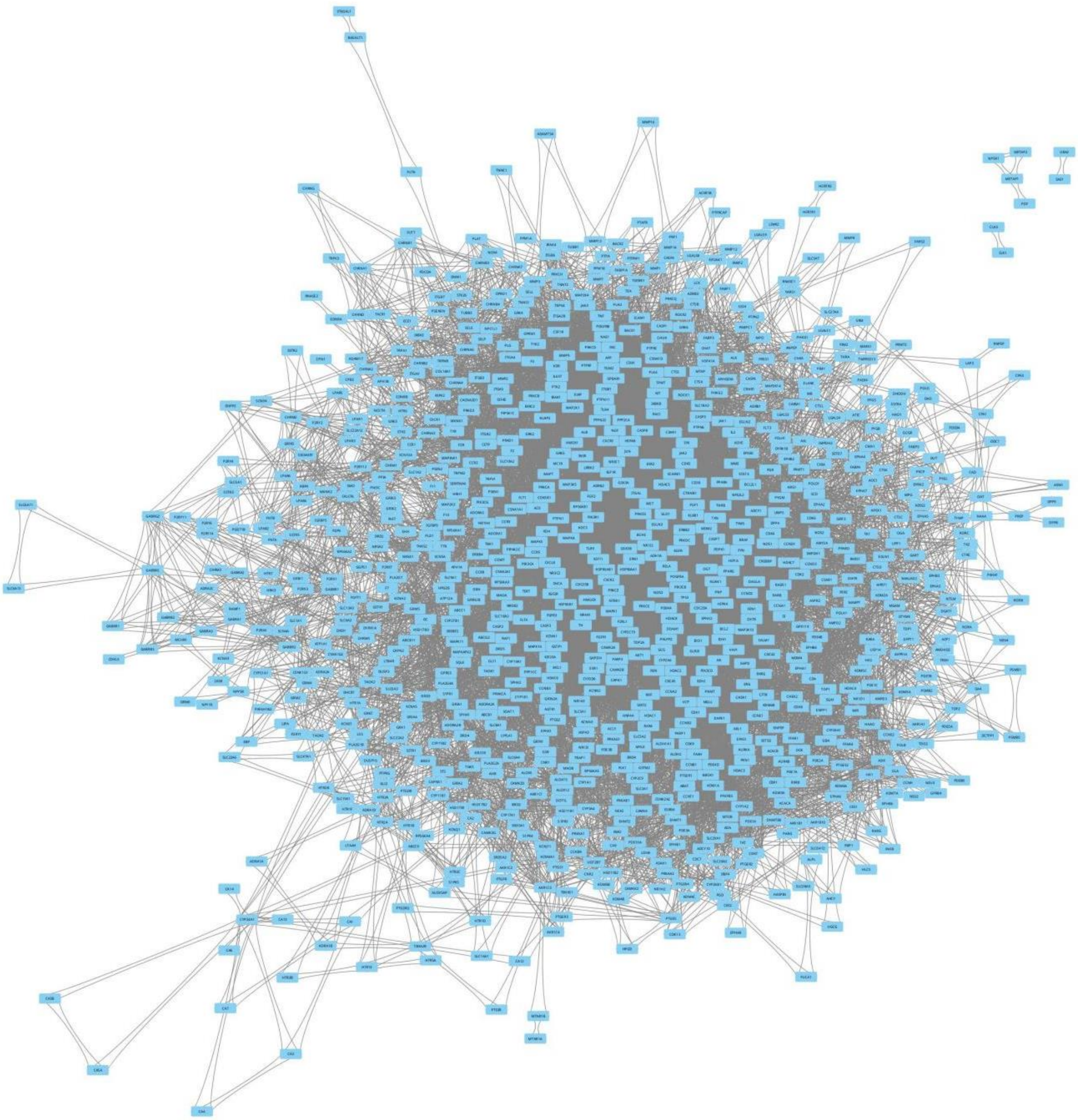

Figure S30: Complete network of CSE target genes

MicroRNA and Target Gene Description:			
**miRNA Name**	hsa-let-7d-5p	**miRNA Sequence**	AGAGGUAGUAGGUUGCAUAGUU
**Previous Name**	hsa-let-7d		
**Target Score**	53	**Seed Location**	316
**NCBI Gene ID**	3569	**GenBank Accession**	NM_000600
**Gene Symbol**	IL6	**3' UTR Length**	437
**Gene Description**	interleukin 6		

3' UTR Sequence

1 catgggcacc tcagattgtt gttgttaatg ggcattcctt cttctggtca gaaacctgtc
61 cactgggcac agaacttatg ttgttctcta tggagaacta aaagtatgag cgttaggaca
121 ctattttaat tatttttaat ttattaatat ttaaatatgt gaagctgagt taatttatgt
181 aagtcatatt tatattttta agaagtacca cttgaaacat tttatgtatt agttttgaaa
241 taataatgga aagtggctat gcagtttgaa tatcctttgt ttcagagcca gatcatttct
301 tggaaagtgt aggct**tacct ca**aataaatg gctaacttat acatattttt aaagaaatat
361 ttatattgta tttatataat gtataaatgg tttttatacc aataaatggc attttaaaaa **(A)**
421 attcagcaaa aaaaaaa

Figure S31: IL6 as a binding target for the miRNA-let 7d. **(A)** IL6 sequence at the 3'UTR domain.

**MicroRNA and Target Gene Description:**			
**miRNA Name**	hsa-let-7d-5p	**miRNA Sequence**	AGAGGUAGUAGGUUGCAUAGUU
**Previous Name**	hsa-let-7d		
**Target Score**	51	**Seed Location**	128
**NCBI Gene ID**	4312	**GenBank Accession**	NM_002421
**Gene Symbol**	MMP1	**3' UTR Length**	528
**Gene Description**	matrix metallopeptidase 1		

3' UTR Sequence

1 acattactaa tttgaatgga aaacacatgg tgtgagtcca aagaaggtgt tttcctgaag
61 aactgtctat tttctcagtc atttttaacc tctagagtca ctgatacaca gaatataatc
121 ttatttta**tac ctca**gtttgc atattttttt actatttaga atgtagccct ttttgtactg
181 atataattta gttccacaaa tggtgggtac aaaaagtcaa gtttgtggct tatggattca
241 tataggccag agttgcaaag atcttttcca gagtatgcaa ctctgacgtt gatcccagag
301 agcagcttca gtgacaaaca tatcctttca agacagaaag agacaggaga catgagtctt
361 tgccggagga aaagcagctc aagaacacat gtgcagtcac tggtgtcacc ctggataggc
421 aagggataac tcttctaaca caaaataagt gttttatgtt tggaataaag tcaaccttgt (**A**)
481 ttctactgtt ttatacactt tcaaaaaaaa aaaaaaaaaa aaaaaaaa

Figure S32: MMP1 as a binding target for hsa-let 7d. (**A**) MMP1 sequence at the 3'UTR domain.

Table S1. The list of metabolites detected in the methanolic extract of CSE by HPLC analysis.

No.	Class	Detected metabolite	Concentration (μg/mL)
**1**	**Phenolic acid**	Catechol	14.52
**2**		Syringic acid	10.69
**3**		Cinnamic acid	6.05
**4**		Caffeic acid	5.12
**5**		Salicylic acid	2.44
**6**		Ferulic acid	1.32
**7**	**Flavonoid**	Quercetin	13.07
**8**		Rutin	10.48
**9**		Luteolin	3.08
**10**		Apigenin	2.13
**11**		Naringin	1.88
**12**		Kaempferol	1.33
**13**	**WSV**	Vitamin B2 (Riboflavin)	10.16
**14**		Vitamin B3 (Niacin)	9.69
**15**		Vitamin B6 (Pyridoxine)	8.36
**16**		Vitamin B1 (Thiamine)	6.14
**17**		Vitamin C (Ascorbic acid)	1.41
**18**	**FSV**	Vitamin K	12.55
**19**		Vitamin D	9.22
**20**		Retinol (Vitamin A)	8.74

Table S2: The list of detected metabolites in *CSE* by UPLC/T-TOF–MS/MS spectrometer in the positive ionization mode.

Title	RT (min)	Precursor (*m/z*)	Area	Error (PPM)	Adduct	Reference (*m/z*)	Formula	Classification
**Spermidine**	0.846	146.1647	19750	0.9	[M+H]+	146.16518	C7H19N3	Dialkylamines
**4-AMINOPHENOL**	0.902	110.0092	111105	-4.9	[M+H]+	110.06004	C6H7NO	Aniline and substituted anilines
**Agmatine**	0.928	131.13	11338	-10.9	[M+H]+	131.12912	C5H14N4	Guanidines
**L-Arginine**	0.953	175.1201	25483	-12.1	[M+H]+	175.11896	C6H14N4O2	L-alpha-amino acids
**N,N-Dimethylglycine**	1.004	104.1072	7232068	-2.9	[M+H]+	104.0706	C4H9NO2	Alpha amino acids
**Maltotriose**	1.091	505.1754	1814235	0	[M+H]+	505.1763	C18H32O16	Oligosaccharides
**Melibiose**	1.116	343.1241	3607872	-0.6	[M+H]+	343.12347	C12H22O11	O-glycosyl compounds
**Xanthosine-5'-monophosphate**	1.116	365.1061	801786	0.5	[M+H]+	365.04929	C10H13N4O9P	Purine ribonucleoside monophosphates
**Glycine-Betaine**	1.128	118.0855	328778	0.8	[M+H]+	118.08626	C5H11NO2	Alpha amino acids
**2'-Deoxyadenosine 5'-monophosphate**	1.166	332.133	72282	-1.5	[M+H]+	332.07544	C10H14N5O6P	Purine 2'-deoxyribonucleoside monophosphates
**3-(4-HYDROXY-3-METHOXYPHENYL)PROP-2-ENOICACID**	1.229	195.1142	94136	-3.2	[M+H]+	195.06519	C10H10O4	Hydroxycinnamic acids
**Tyr**	1.242	182.0777	19539	0.1	[M+H]+	182.08118	C9H11NO3	Tyrosine and derivatives
**Resveratrol**	1.267	229.1424	11088	42.8	[M+H]+	229.08592	C14H12O3	Stilbenes
**3-FORMYLINDOLE**	1.280	146.0605	27786	-6.7	[M+H]+	146.06004	C9H7NO	Indoles
**Scoulerin**	1.330	328.1689	216477	16.5	[M+H]+	328.15433	C19H21NO4	Protoberberine alkaloids and derivatives
**NICOTINIC ACID**	1.441	124.0393	122323	0.9	[M+H]+	124.03931	C6H5NO2	Pyridinecarboxylic acids
**L-5-Oxoproline**	1.678	130.0508	63193	-9.4	[M+H]+	130.04987	C5H7NO3	Alpha amino acids and derivatives
**Adenosine**	1.727	268.1041	186403	2	[M+H]+	268.10403	C10H13N5O4	Purine nucleosides
**Adenosine 5'-monophosphate**	1.837	348.197	88105	40.8	[M+H]+	348.07037	C10H14N5O7P	Purine ribonucleoside monophosphates
**Adenosine 3':5'-cyclicmonophosphate**	2.313	330.1187	65290	1.1	[M+H]+	330.05978	C10H12N5O6P	3',5'-cyclic purine nucleotides
**L-TRYPTOPHAN**	2.748	205.0975	1248671	-0.6	[M+H]+	205.09715	C11H12N2O2	Indolyl carboxylic acids and derivatives
**Cytidine 5'-**	4.474	489.1373	61304	0.5	[M+H]+	489.11459	C14H26N4O11	Pyrimidine ribonucleoside

**diphosphocholine**							P2	diphosphates
**Procyanidin B1**	4.535	579.1257	11328	-29.6	[M+H]+	579.14972	C30H26O12	Biflavonoids and polyflavonoids
**CHOLIC ACID**	4.779	409.1111	36907	0.1	[M+H]+	409.29486	C24H40O5	Trihydroxy bile acids, alcohols and derivatives
**Luteolin-3', 7-di-O-glucoside**	4.950	611.1547	10863	1.3	[M+H]+	611.16064	C27H30O16	Flavonoid-7-O-glycosides
**Quercetin**	4.962	303.0485	21648	-3.8	[M+H]+	303.04993	C15H10O7	Flavonols
**Quercetin-7-O-rhamnoside**	4.962	449.1088	50703	-0.2	[M+H]+	449.10785	C21H20O11	Flavonoid-7-O-glycosides
**Kaempferol-3-O-alpha-L-rhamnoside**	5.244	433.1106	144655	5.4	[M+H]+	433.11292	C21H20O10	Flavonoid-3-O-glycosides
**Luteolin**	5.256	287.0526	47957	2.7	[M+H]+	287.05502	C15H10O6	Flavones
**Trigonelline**	5.748	138.0548	232065	0.4	[M+H]+	138.05496	C7H7NO2	Alkaloids and derivatives
**Delphinidin-3-O-(6''-O-alpha-rhamnopyranosyl-beta-glucopyranoside)**	5.870	611.1556	116294	7.9	[M]+	611.1601	C27H31O16	Anthocyanidin-3-O-glycosides
**Riboflavin-5?-monophosphate sodium salt hydrate**	6.127	457.1563	10059	1.4	[M+H]+	457.11188	C17H21N4O9P	Flavin nucleotides
**cyanidin-3-O-rutinoside**	6.264	595.1705	84546	-2.2	[M]+	595.16522	C27H31O15	Anthocyanidin-3-O-glycosides
**Isorhamnetin-3-O-rutinoside**	6.486	625.1713	59691	2.5	[M+H]+	625.17633	C28H32O16	Flavonoid-3-O-glycosides
**5-Aminoimidazole-4-carboxamide-1-ribofuranosyl 5'-monophosphate**	7.193	339.1373	48121	23.2	[M+H]+	339.07004	C9H15N4O8P	1-ribosyl-imidazolecarboxamides
**3-(4-HYDROXY-3,5-DIMETHOXYPHENYL)-2-PROPENOIC ACID**	7.282	225.0738	41205	0.2	[M+H]+	225.07574	C11H12O5	Hydroxycinnamic acids
**Sinapoyl malate**	8.000	341.0876	634340	-0.2	[M+H]+	341.0867	C15H16O9	Coumaric acids and derivatives
**Acacetin-7-O-rutinoside**	9.296	593.184	24221	2.3	[M+H]+	593.18646	C28H32O14	Flavonoid-7-O-glycosides
**(-)-RIBOFLAVIN**	9.576	377.0845	52970	0.7	[M+H]+	377.14557	C17H20N4O6	Flavins
**Acacetin-7-O-neohesperidoside**	10.138	593.3146	201047	-0.1	[M+H]+	593.18646	C28H32O14	Flavonoid-7-O-glycosides

**Guanosine 5'-diphosphate-D-mannose**	10.307	606.2593	12228	-0.8	[M+H]+	606.08441	C16H25N5O16 P2	Purine nucleotide sugars
**D-Glucosamine-6-phosphate**	10.670	260.0636	14527	7.6	[M+H]+	260.05298	C6H14NO8P	Hexose phosphates
**Maritimetin-6-O-glucoside**	11.023	449.16	50939	3.2	[M+H]+	449.10785	C21H20O11	Aurone O-glycosides
**Anserine**	11.639	241.1428	61283	0	[M+H]+	241.12952	C10H16N4O3	Hybrid peptides
**Gossypin**	12.699	481.1161	48521	-1.5	[M+H]+	481.09766	C21H20O13	Flavonoid-8-O-glycosides
**N,N-Dimethylformamide**	15.056	74.09587	37691	3.7	[M+H]+	74.06004	C3H7NO	Tertiary carboxylic acid amides
**3' 4' 5 7-tetrahydroxyflavanone**	18.007	289.1549	16777	0.3	[M+H]+	289.07068	C15H12O6	Flavanones
**3'-METHOXY-4',5,7-TRIHYDROXYFLAVONOL**	19.675	317.1152	750110	-0.4	[M+H]+	317.06558	C16H12O7	Flavonols
**D-(-)-Erythrose**	19.691	121.0267	84673	-1.4	[M+H]+	121.04954	C4H8O4	Pentoses
**2'-Deoxycytidine**	19.936	228.231	866922	5.9	[M+H]+	228.09789	C9H13N3O4	Pyrimidine 2'-deoxyribonucleosides
**Isoguvacine**	21.443	128.1048	32494	4.4	[M+H]+	128.0706	C6H9NO2	Hydropyridines
**3 5 7-trihydroxy-4'-methoxyflavone**	21.506	301.1419	630989	0.7	[M+H]+	301.07068	C16H12O6	Flavonols
**Formononetin**	22.107	269.0993	20083	0.5	[M+H]+	269.08084	C16H12O4	4'-O-methylisoflavones
**Leupeptin hemisulfate salt**	24.166	427.3768	6502	9.1	[M+H]+	427.30273	C20H38N6O4	Dipeptides
**Choline**	26.811	104.1057	6708	6.9	[M]+	104.10645	C5H14NO	Cholines

Table S3: The list of detected metabolites in *C. endivia* extract by UPLC/T-TOF–MS/MS spectrometer in the negative ionization mode.

Title	RT (min)	Precursor (*m/z*)	Area	Error (PPM)	Adduct	Reference (*m/z*)	Formula	Classification
**Citrate**	0.862	191.0182	40416	3.7	[M-H]-	191.01973	C6H8O7	Tricarboxylic acids and derivatives
**D-(+)-Malic acid**	0.862	133.0122	117239	7	[M-H]-	133.01425	C4H6O5	Beta hydroxy acids and derivatives
**Sorbitol 6-phosphate**	0.862	260.9597	9633	-1.3	[M-H]-	261.03809	C6H15O9P	Monosaccharide phosphates
**Gluconate**	0.927	195.0501	164879	1.1	[M-H]-	195.05103	C6H12O7	Medium-chain hydroxy acids and derivatives
**Inosine**	0.927	267.0702	90934	4	[M-H]-	267.07349	C10H12N4O 5	Purine nucleosides
**L-Glutamic acid**	0.927	146.0436	42186	8.5	[M-H]-	146.04588	C5H9NO4	Glutamic acid and derivatives
**HydroxyButyric acid**	0.941	102.9552	21977	4.9	[M-H]-	103.04007	C4H8O3	Alpha hydroxy acids and derivatives
**Mucate**	0.941	209.0661	36575	0.1	[M-H]-	209.03029	C6H10O8	Glucuronic acid derivatives
**L-5-Oxoproline**	0.954	128.0348	122085	-0.4	[M-H]-	128.03532	C5H7NO3	Alpha amino acids and derivatives
**L-beta-Homolysine**	0.967	159.0297	24064	-2.8	[M-H]-	159.11391	C7H16N2O2	Beta amino acids and derivatives
**2,5-DIHYDROXYBENZOIC ACID**	0.994	153.0184	25745	3.9	[M-H]-	153.01933	C7H6O4	Hydroxybenzoic acid derivatives
**3-(4-HYDROXY-3-METHOXYPHENYL)PROP-2-ENOICACID**	1.007	193.0498	29718	-0.3	[M-H]-	193.05063	C10H10O4	Hydroxycinnamic acids
**3-(4-HYDROXY-3,5-DIMETHOXYPHENYL)-2-PROPENOIC ACID**	1.020	223.0624	815302	-5	[M-H]-	223.0612	C11H12O5	Hydroxycinnamic acids
**L-(+)-Tartrate**	1.020	149.025	4730	131.7	[M-H]-	149.00916	C4H6O6	Sugar acids and derivatives
**Sinapoyl malate**	1.020	339.0699	1667280	6.1	[M-H]-	339.07214	C15H16O9	Coumaric acids and derivatives
**D-(+)-Raffinose**	1.057	503.162	3052157	0.2	[M-H]-	503.16177	C18H32O16	Oligosaccharides
**Daidzein-8-C-glucoside**	1.057	415.1393	94078	12.8	[M-H]-	415.10345	C21H20O9	Isoflavonoid C-glycosides
**Pantothenate**	1.070	218.1017	12291	1.9	[M-H]-	218.10339	C9H17NO5	Secondary alcohols
**MANNITOL**	1.108	181.0717	92335	1	[M-H]-	181.07176	C6H14O6	Sugar alcohols

**Sucrose**	1.108	341.1086	8841210	0.9	[M-H]-	341.10895	C12H22O11	O-glycosyl compounds
**3'-METHOXY-4',5,7-TRIHYDROXYFLAVONOL**	1.133	315.0705	478035	2.3	[M-H]-	315.05103	C16H12O7	Flavonols
**Xanthosine-5'-monophosphate**	1.145	363.0856	186672	8.1	[M-H]-	363.03473	C10H13N4O9P	Purine ribonucleoside monophosphates
**2'-Deoxyuridine-5'-monophosphate**	1.208	307.0807	69680	5.2	[M-H]-	307.03366	C9H13N2O8P	Pyrimidine 2'-deoxyribonucleoside monophosphates
**E-3,4,5'-Trihydroxy-3'-glucopyranosylstilbene**	1.208	405.1152	55281	-0.3	[M-H]-	405.11911	C20H22O9	Stilbene glycosides
**Kaempferol-3-Glucuronide**	1.208	461.1288	429942	3.9	[M-H]-	461.07254	C21H18O12	Flavonoid-3-O-glucuronides
**p-Hydroxybenzylglucosinolate**	1.208	424.0347	36240	4.7	[M-H]-	424.03775	C14H19NO10S2	Alkylglucosinolates
**2'-Deoxyuridine**	1.246	227.1304	26293	-6.7	[M-H]-	227.06735	C9H12N2O5	Pyrimidine 2'-deoxyribonucleosides
**Benzyl glucosinolate**	1.483	408.0423	38789412	0.3	[M-H]-	408.04285	C14H19NO9S2	Alkylglucosinolates
**L-(-)-Phenylalanine**	1.992	164.0757	14225	-13.6	[M-H]-	164.0717	C9H11NO2	Phenylalanine and derivatives
**L-TRYPTOPHAN**	2.602	203.0799	181671	9.8	[M-H]-	203.0826	C11H12N2O2	Indolyl carboxylic acids and derivatives
**2'-Deoxyuridine-5'-triphosphate sodium salt**	5.168	467.1586	381791	0.6	[M-H]-	466.96634	C9H15N2O14P3	Pyrimidine 2'-deoxyribonucleoside triphosphates
**1-O-b-D-glucopyranosyl sinapate**	5.231	385.1152	161472	-1.1	[M-H]-	385.11401	C17H22O10	Hydroxycinnamic acid glycosides
**Cytidine-5'-diphosphate**	5.345	402.0727	20022	5.9	[M-H]-	402.01089	C9H15N3O11P2	Pyrimidine ribonucleoside diphosphates
**Luteolin-3', 7-di-O-glucoside**	5.805	609.1465	49060	-0.4	[M-H]-	609.14612	C27H30O16	Flavonoid-7-O-glycosides
**3-Indoxyl sulfate**	5.818	212.0004	388017	7.3	[M-H]-	212.0023	C8H7NO4S	Arylsulfates
**Kaempferol-3-O-(6-p-coumaroyl)-glucoside**	6.130	593.1541	139778	-3.6	[M-H]-	593.13007	C30H26O13	Flavonoid 3-O-p-coumaroyl glycosides
**Eriodictyol-7-O-**	6.674	595.1813	17958	0.6	[M-H]-	595.16687	C27H32O15	Flavonoid-7-O-glycosides

**neohesperidoside**								
**Okanin-4'-O-glucoside**	6.699	449.1491	189077	0.3	[M-H]-	449.10895	C21H22O11	Flavonoid O-glycosides
**Procyanidin B2**	6.724	577.1213	29996	8.9	[M-H]-	577.13513	C30H26O12	Biflavonoids and polyflavonoids
**eriodictyol-7-O-glucoside**	6.966	449.1434	44691	10	[M-H]-	449.10895	C21H22O11	Flavonoid-7-O-glycosides
**Quercetin-3,4'-O-di-beta-glucopyranoside**	7.068	625.187	42346	-0.8	[M-H]-	625.14105	C27H30O17	Flavonoid-3-O-glycosides
**Rhoifolin**	7.309	577.1554	9240	0.8	[M-H]-	577.15625	C27H30O14	Flavonoid-7-O-glycosides
**L-beta-Homoisoleucine**	8.018	144.045	27281	0.2	[M-H]-	144.103	C7H15NO2	Beta amino acids and derivatives
**2'-Deoxyinosine 5'-monophosphate**	10.097	330.9788	106333	1	[M-H]-	331.04492	C10H13N4O7P	Purine 2'-deoxyribonucleoside monophosphates
**Apigenin**	10.347	269.0478	27422	-0.2	[M-H]-	269.04553	C15H10O5	Flavones
**Sinapyl aldehyde**	10.634	207.0659	30139	-0.6	[M-H]-	207.06628	C11H12O4	Methoxyphenols
**gamma-Linolenic acid**	11.093	277.1809	31517	-0.8	[M-H]-	277.21732	C18H30O2	Lineolic acids and derivatives
**Esculin**	15.752	339.2023	35905	-4.2	[M-H]-	339.07214	C15H16O9	Coumarin glycosides
**Gibberelin A3**	19.063	345.2	90314	4.3	[M-H]-	345.13437	C19H22O6	C19-gibberellin 6-carboxylic acids
**rosmarinic acid**	22.130	359.1499	691351	3.6	[M-H]-	359.07724	C18H16O8	Coumaric acids and derivatives

Table S8: **The list of lncRNAs that are predicted to interact with the microRNA hsa-let-7d-5p.** lncRNAs predicted by DIANA-lncBase v3.0 to interact with hsa-let-7d-5p are listed with their corresponding miTG scores. All predictions show high confidence (miTG > 0.99).

Transcript_ID	Gene_ID (Gene_Name)	Mirna_Name (miRBase_version)	miTG-score
**ENST00000623644**	ENSG00000279978(chr22-38_28785274-29006793.1)	hsa-let-7d-5p(21)	0.999999453
**ENST00000602324**	ENSG00000269959(SPACA6P-AS)	hsa-let-7d-5p(21)	0.999997963
**ENST00000618634**	ENSG00000278451(RP11-923I11.8)	hsa-let-7d-5p(21)	0.999670428
**TCONS_00026972**	XLOC_013024(XLOC_013024)	hsa-let-7d-5p(21)	0.999107857
**ENST00000588424**	ENSG00000100181(TPTEP1)	hsa-let-7d-5p(21)	0.998573718
**TCONS_00017944**	XLOC_008765(XLOC_008765)	hsa-let-7d-5p(21)	0.99698722
**TCONS_00028843**	XLOC_013931(XLOC_013931)	hsa-let-7d-5p(21)	0.994777894
**ENST00000597346**	ENSG00000269821(KCNQ1OT1)	hsa-let-7d-5p(21)	0.992461749
**ENST00000580134**	ENSG00000265494(RP11-131K5.2)	hsa-let-7d-5p(21)	0.990596154

Table S9: The list of gene primers utilized for reverse transcription-quantitative polymerase chain reactions.

Reaction type	Primer	Sequences 5′-3′
**RT-PCR**	miR-let 7d 5p (F)	GGCATGGACGAGCTGTCAA
	miR-let 7d 5p (R)	CTCTAGATCAACCACTTTGT
	ncNRFR (F)	CAAGGATAAAGCTTGTGTGGTT
	ncNRFR (R)	AGAGGTAGTAGGTTGCATAGTT
	GAPDH (F)	GGAGCGAGATCCCTCCAAAAT
	GAPDH (R)	GGCTGTTGTCATACTTCTCATGG
	U6(F)	CTCGCTTCGGCAGCACA
	U6(R)	AACGCTTCACGAATTTGCGT
